# Ulcerative colitis-driven gut dysbiosis exacerbates periodontal bone loss through the gut–oral axis /Th17/Treg imbalance

**DOI:** 10.1038/s41522-026-01015-6

**Published:** 2026-05-23

**Authors:** Yue Huang, Yu Hu, Yifei Zhao, Yuerui Li, Yunkun Liu, Miao Wang, Qian Wang, Huan Hu

**Affiliations:** 1 School of Stomatology, Zunyi Medical University, Zunyi, 563000 China; 2 Key Laboratory of Microbial Resources and Drug Development of Guizhou Education Department, Zunyi Medical University, Zunyi, 563000 China; 3 Key Laboratory of Oral Disease Research of Guizhou Education Department, Zunyi Medical University, Zunyi, 563000 China

**Keywords:** Diseases, Microbiology

## Abstract

Ulcerative colitis (UC) and periodontitis, both microbial dysbiosis-driven chronic inflammatory disorders, coexist and mutually exacerbate, but the causal mechanisms remain unclear. Using ligature-induced periodontitis plus DSS-colitis mice, we found UC doubles alveolar bone loss, heightens systemic inflammation, oxidative stress, and osteoclastogenesis. 16S rRNA and LC–MS metabolomics showed UC enriches oral pathogens, depletes gut Firmicutes, expands Bacteroides, and correlates with suppressed amino-acid/bile-acid biosynthesis. Fecal microbiota transplantation (FMT) from DSS donors into antibiotic-pretreated periodontitis-prone mice replicated aggravated bone loss, systemic inflammation, gut-barrier leakage, and Th17/Treg imbalance, while healthy-donor FMT protected. GC–MS revealed 35–60% reductions in acetate, propionate, and butyrate; keystone taxa *Parabacteroides* and *Muribaculum* inversely correlated with SCFAs and host inflammatory genes. Collectively, UC-driven gut dysbiosis is a transmissible causal factor that simultaneously remodels oral and intestinal biofilms, erodes epithelial barriers, and amplifies osteoclastic bone resorption. SCFAs-producing microbes or supplementation may be potential therapeutics for UC-associated periodontitis patients.

**Figure:**
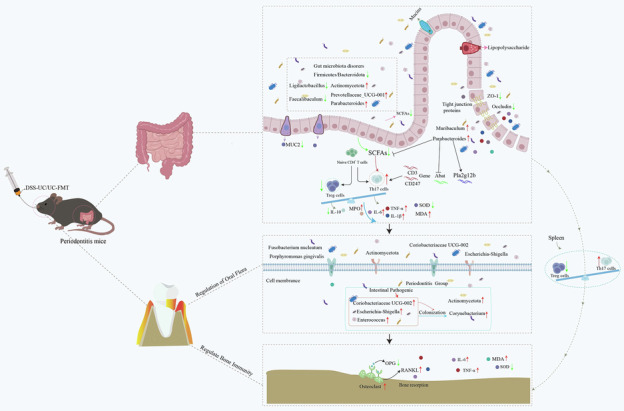

## Introduction

Periodontitis is one of the most common chronic inflammatory diseases of humans and is characterized by progressive destruction of the tooth-supporting tissues, culminating in irreversible alveolar bone loss and eventual tooth exfoliation. The disease is initiated by dysbiotic subgingival biofilms, but tissue breakdown is executed by host-derived osteoclasts and matrix-degrading proteases that are hyper-activated by an exaggerated local and systemic immune response. Key molecular signatures of active periodontitis, therefore, include elevated receptor activator of NF-κB ligand (RANKL), pro-inflammatory cytokines such as interleukin-1β (IL-1β), interleukin-6 (IL-6), and tumor necrosis factor-α (TNF-α), and the expansion of Th17 cells that further amplify neutrophil recruitment and osteoclastogenesis^1^. Inflammatory bowel disease (IBD), encompassing Crohn’s disease (CD) and ulcerative colitis (UC), is another relapsing-remitting inflammatory disorder in which similar immunological pathways drive mucosal damage. The pathogenesis of IBD is intricately linked to host genetic susceptibility, intestinal dysbiosis, intestinal barrier impairment, and aberrations in intestinal mucosal immunity^2^. Recent epidemiological surveys have consistently elucidated that IBD patients exhibit a higher prevalence and severity of periodontitis than matched controls, even after adjustment for oral hygiene^3,4^. Yet, whether the intestinal inflammation itself is sufficient to exacerbate periodontal bone loss, and through which mechanistic axis, remains undefined. This emerging recognition highlights the necessity for further investigating the interrelations between these two inflammatory conditions, which may offer novel insights into their clinical management and therapeutic strategies.

Trillions of symbiotic microorganisms colonizing the oral cavity and intestines play a vital role in reflecting and regulating human health. They maintain homeostasis through continuous crosstalk among the microbiome, the host, and intra-microbial components, thereby suppressing the overgrowth of pathogenic microbes^5^. The human body harbors distinct yet interconnected microbial consortia along the digestive tract. Physical and chemical barriers—including gastric acid, bile salts, gastrointestinal mucosal barriers and sphincters—largely restrict translocation, but these defenses can be breached during inflammation, iatrogenic acid suppression or aging^6,7^. Culture-independent sequencing has repeatedly detected oral taxa—especially *Fusobacterium nucleatum*, *Porphyromonas gingivalis*, and *Prevotella spp*.—within colonic biopsies and feces of IBD patients, where their abundance correlates with systemic pro-inflammatory level and endoscopic severity scores^8^. Animal studies show that oral administration of *F. nucleatum* accelerates DSS-induced colitis via FadA-mediated adherence to E-cadherin and subsequent β-catenin signaling^9,10^. Additionally, oral inoculation of *P. gingivalis* accelerates DSS-colitis via suppressing gut microbiota-linoleic acid metabolism and disrupting the Th17/Treg cell balance axis, thereby reinforcing the concept of an oro-intestinal microbial axis^11^. However, whether IBD-associated systemic inflammation alters the oral microbiome, leading to oral dysbiosis and exacerbated oral inflammation, remains to be fully elucidated.

From an immunological perspective, the digestive tract serves as the primary interface between the internal and external environments of the body and acts as a crucial barrier against pathogen invasion. Host defense mechanisms against such invasions encompass both innate and adaptive immunity, with T lymphocytes playing a central role in immune defense. Specifically, CD4^+^ T lymphocytes patrol both oral and intestinal compartments and display plastic surface markers that facilitate compartmental homing^12–14^. During periodontitis, bacterial virulence factors in dysbiotic plaque prime oral dendritic cells, which preferentially polarize naïve CD4 T cells toward IL-17-secreting Th17 cells that express the integrin α4β7. Ligands for α4β7—mucosal addressin cell-adhesion molecule-1 (MAdCAM-1) and CCL25—are markedly upregulated in the inflamed ileum and colon, providing a molecular highway for oral Th17 cells to migrate to the gut and perpetuate intestinal inflammation^15,16^. Furthermore, several investigations have explored whether IBD influences periodontitis via immune-mediated pathways by assessing the expression levels of various cytokines. For instance, cytokine measurements in saliva and gingival crevicular fluid of IBD patients reveal elevated TNF-α, IL-1β, IL-6, interleukin-21 (IL-21), and interferon-γ (IFN-γ), whose concentrations scale with both mucosal and periodontal disease activity^17,18^. These observations suggest that immune cells and cytokines may constitute a common currency in the IBD-periodontitis dialog, yet direct evidence that intestinal inflammation is the initiating driver of periodontal bone destruction is still lacking.

Beyond cells and cytokines, microbial metabolites act as long-range signaling molecules that couple the gut to distant organs. Short-chain fatty acids (SCFAs)—principally acetate, propionate, and butyrate—are generated by fiber-fermenting Firmicutes and Bacteroidetes and maintain intestinal barrier integrity, induce colonic Treg differentiation, and restrain systemic osteoclastogenesis through HDAC-inhibition and GPR43/109 A signaling^19–21^. The intestinal microbiota serves as a crucial medium for maintaining bone homeostasis and has been shown to influence alveolar bone homeostasis through various mechanisms, including the regulation of intestinal barrier integrity, modulation of the intestinal immune response, and systemic endocrine metabolism^22^. IBD-associated dysbiosis consistently reduces fecal SCFA concentrations, and oral butyrate supplementation ameliorates experimental colitis^23^. Whether SCFA depletion is equally operative in periodontal bone loss has not been explored. We then employed antibiotic-mediated microbiota depletion and reciprocal fecal microbiota transplantation (FMT) to establish causality, showing that a UC-associated microbiome is both necessary and sufficient to transmit the periodontal phenotype. Through this comprehensive approach, we seek to provide novel insights into the interplay between colitis and periodontitis, advancing our understanding of their underlying pathogenic mechanisms.

In this study, we hypothesized that UC-driven gut dysbiosis exacerbates periodontal bone loss through the gut–oral axis, involving immune dysregulation and microbial metabolic disturbances. To test this hypothesis, we developed a comorbid mouse model of DSS-induced experimental UC and ligature-induced periodontitis, and employed antibiotic-mediated microbiota depletion coupled with reciprocal FMT to establish causality. Our findings demonstrated that UC-associated gut dysbiosis is both necessary and sufficient to transmit the periodontal phenotype, providing mechanistic insights into the interplay underlying the colitis-periodontitis comorbidity and highlighting potential targets for integrated therapeutic interventions.

## Results

### UC amplifies periodontal pathogenesis via systemic inflammation, microbiota remodeling, and metabolomic shifts

#### UC aggravates systemic inflammation, oxidative stress, and alveolar bone resorption in mice with periodontitis

**Fig. 1:**
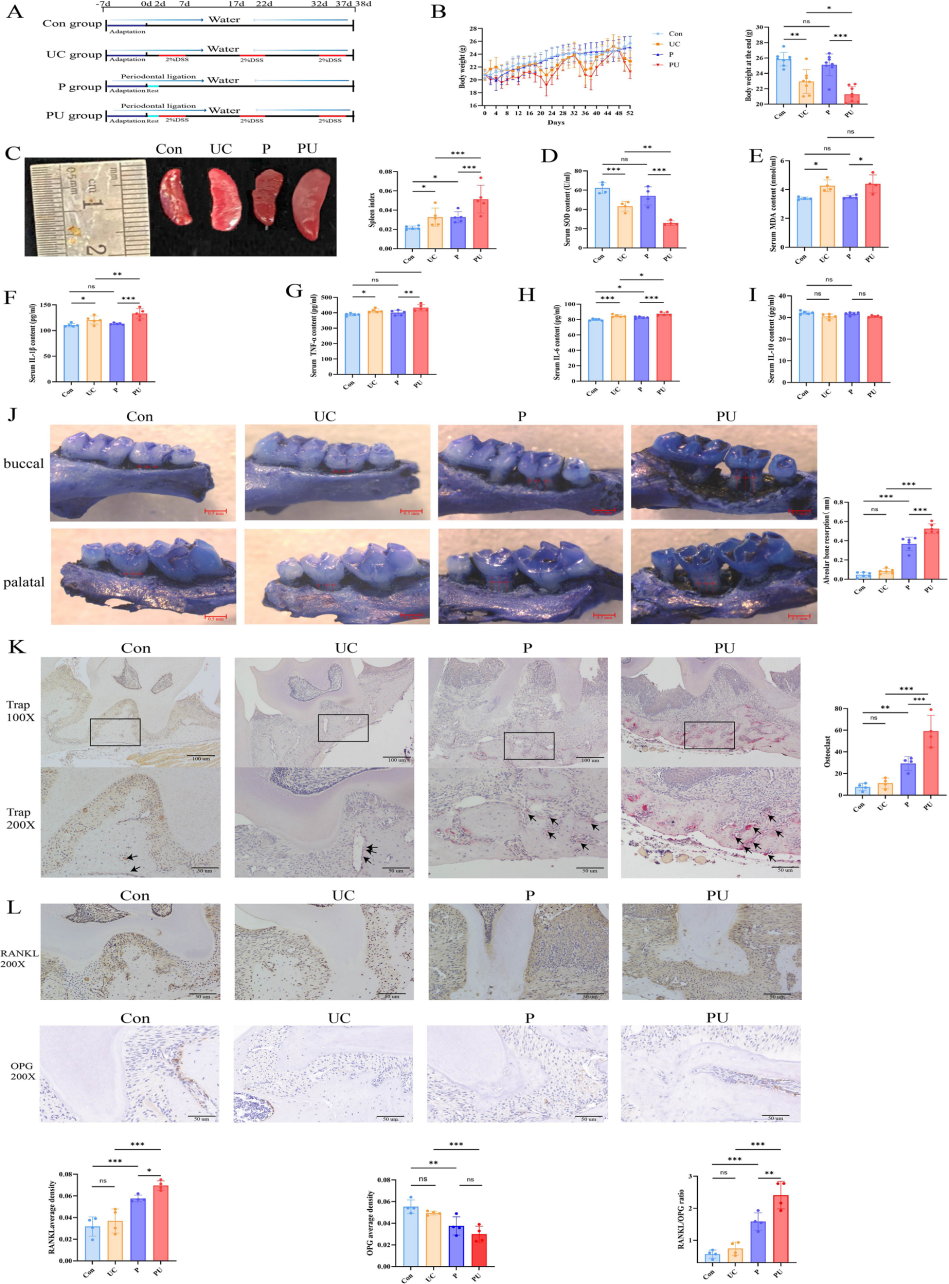
UC aggravates systemic inflammation, oxidative stress, and alveolar bone resorption in mice with periodontitis. **A** Experimental design: Periodontitis was induced by ligating the second maxillary molar (5-0 silk ligature); ulcerative colitis (UC)-like colitis was induced by 2% dextran sulfate sodium (DSS) in drinking water (5 days DSS/10 days water cycles, total 35 days). **B** Body weight changes (*N* = 8/group). **C** Spleen index (*N* = 5/group). **D**, **E** Serum superoxide dismutase (SOD) activity and malondialdehyde (MDA) levels (*N* = 4/group). **F**–**I** Serum pro-inflammatory cytokines interleukin-1β (IL-1β), interleukin-6 (IL-6), tumor necrosis factor-α (TNF-α), and anti-inflammatory cytokine IL-10 (*N* = 5/group). **J** Stereomicroscopic images and quantitative analysis of alveolar bone resorption (*N* = 6/group, Scale bar: 0.5 mm). **K** Representative images of tartrate-resistant acid phosphatase (TRAP)-positive multinucleated osteoclasts in periodontal tissues below the furcation and near the root apex of the maxillary second molar, with corresponding quantification of TRAP-positive multinucleated (≥3 nuclei) osteoclasts per unit area (captured at 100× and 200× magnification, *N* = 4/group, Scale bar: 100 μm and 50 μm). **L** Representative Immunohistochemical staining images showing receptor activator of nuclear factor-κB ligand (RANKL) and osteoprotegerin (OPG) in alveolar bone adjacent to the root surface of the maxillary second molar, and RANKL/OPG ratio with corresponding quantitative analysis of integrated optical density (IOD) (*N* = 4/group, Scale bar: 50 μm). Data are expressed as mean ± standard error of the mean (SEM). *P < 0.05; **P < 0.01; ***P < 0.001.

Simultaneous DSS-induced colitis and ligature-induced periodontitis (PU group) produced a marked systemic inflammatory spike compared with either disease alone (UC or P group, Fig. 1). Mice exhibited accelerated weight loss (Fig. 1B), splenomegaly (higher spleen index, Fig. 1C). Serum redox balance shifted toward oxidative stress: SOD activity fell while MDA rose (Fig. 1D, E). Circulating pro-inflammatory cytokines IL-6, IL-1β, and TNF-α were all significantly elevated (Fig. 1F–H), whereas the anti-inflammatory factor interleukin-10 (IL-10) remained unchanged (Fig. 1I).

Alveolar bone destruction is tightly coupled with the systemic inflammatory phenotype. Stereomicroscopy revealed the most pronounced reduction in alveolar crest height—and thus the greatest extent of bone resorption—in PU mice (Fig. 1J, Figure S1A). Histomorphometric analysis demonstrated a concomitant and significant increase in TRAP-positive osteoclasts within periodontal tissues (Fig. 1K) and a marked upregulation of RANKL protein expression, whereas OPG expression showed a downward trend without reaching statistical significance, resulting in a significant increase in the RANKL/OPG ratio (Fig. 1L). These findings indicate that colitis exacerbates alveolar bone loss by elevating the local RANKL/OPG ratio, thereby promoting osteoclastogenesis and activation specifically within the periodontium.

#### UC remodels the oral microbiota in periodontitis-affected mice

**Fig. 2:**
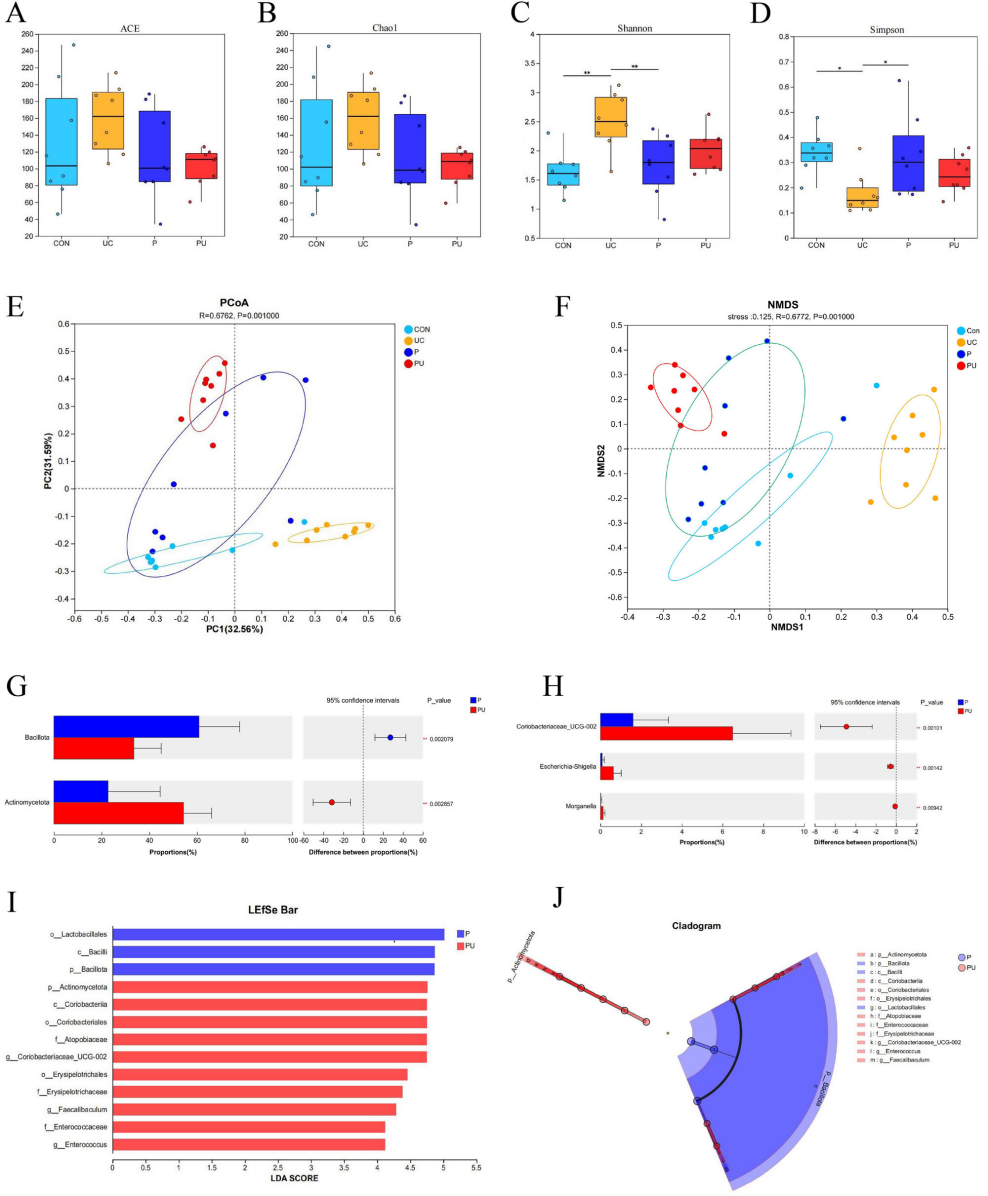
UC remodels the oral microbiota in periodontitis-affected mice (*N* = 8/group). **A**–**D** Alpha diversity indices (ACE, Chao1, Shannon, and Simpson) in the Con, UC, P, and PU groups. **E** Principal coordinate analysis (PCoA) of oral microbiota based on Bray–Curtis distance. **F** Non-metric multidimensional scaling (NMDS) analysis (*R* > 0 indicates inter-group differences exceed intra-group variation). **G**, **H** Relative abundance of bacteria at the phylum (**G**) and genus (**H**) levels in the P and PU groups. **I** Linear discriminant analysis effect size (LEfSe) reveals biomarkers (LDA score > 4) of the P and PU groups. **J** Cladogram of LEfSe multi-level species discriminant analysis (LDA > 4), where different colored nodes indicate microbial communities that are significantly enriched in the corresponding groups and markedly different between groups.

16S rRNA sequencing of saliva was performed to determine whether intestinal inflammation reshapes the oral microbial community. Alpha-diversity metrics (ACE, Chao1, Shannon, and Simpson) did not differ between P and the PU groups (Fig. 2A–D), suggesting that richness and evenness were preserved. Beta-diversity (PCoA and NMDS) nevertheless separated PU samples from both Con and P clusters (Fig. 2E, F), indicating a disease-specific compositional shift.

At the phylum level, *Actinomycetota* was selectively enriched in PU mice (Fig. 2G). Genus-level analysis revealed significant expansion of *Coriobacteriaceae UCG-002*, *Escherichia-Shigella*, and *Morganella* (Fig. 2H). LEfSe coupled to LDA score > 4 identified *Coriobacteriaceae_UCG-002, Faecalibaculum*, and *Enterococcus* as the most discriminatory taxa in the PU group (Fig. 2I, J).

PICRUSt2-based functional inference (KEGG level-3) showed that the top 10 enriched pathways in PU mice included metabolic pathways, biosynthesis of secondary metabolites, microbial metabolism in diverse environments, biosynthesis of amino acids, and ABC transporters. Ribosome, carbon metabolism, purine metabolism, quorum sensing, and starch and sucrose metabolism (Fig. S1B), implying a metabolic shift toward amino-acid scavenging and inter-species signaling.

#### UC drives intestinal dysbiosis and metabolomic rewriting in periodontitis mice

**Fig. 3:**
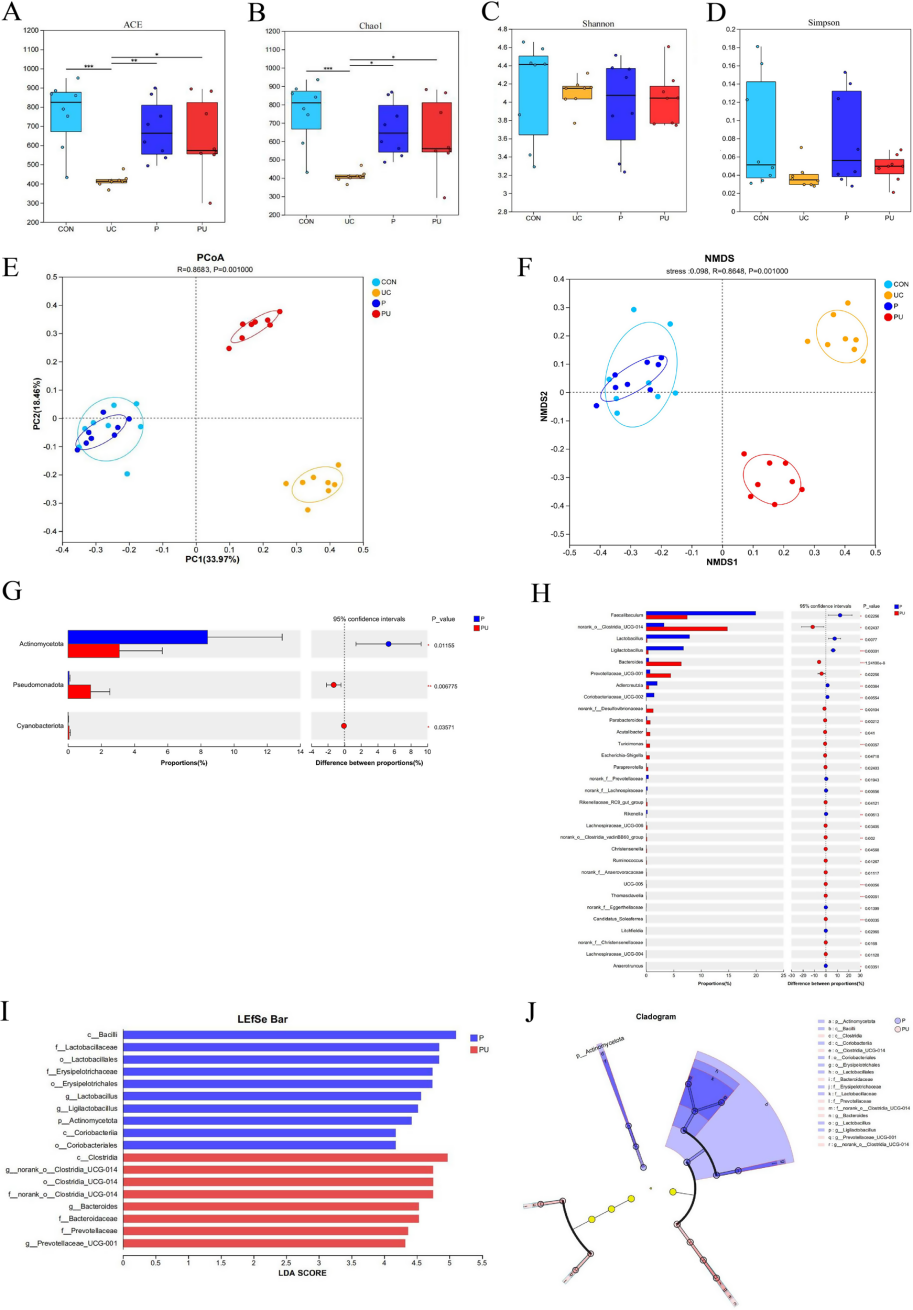
UC remodels the gut microbiota in periodontitis-affected mice (N = 8/group). **A**–**D** Alpha diversity indices (ACE, Chao1, Shannon, and Simpson) in the Con, UC, P, and PU groups. **E** Gut microbiota profiles were examined by PCoA based on Bray–Curtis distance. **F** NMDS analysis (*R* > 0 indicates inter-group differences exceed intra-group variation). **G**, **H** Relative abundance of bacteria at the phylum (**G**) and genus (**H**) levels in the P and PU groups. **I** LEfSe analysis showing biomarkers of the P and PU groups, with taxa exhibiting an LDA > 4 highlighted. **J** Cladogram of LEfSe multi-level species discriminant analysis (LDA > 4), where different colored nodes indicate microbial communities that are significantly enriched in the corresponding groups and markedly different between groups.

Fecal 16S rRNA sequencing was carried out to examine the gut microbiota. Alpha-diversity indices were comparable between P and PU group (Fig. 3A–D), yet PCoA and NMDS plots displayed a clear separation, indicating that UC restructures the community composition of mice with periodontitis (Fig. 3E-F).

Taxonomic differences were evident at both phylum and genus levels. At the phylum level, *Pseudomonadota*, and *Cyanobacteriota* increased, while *Actinomycetota* decreased in PU mice (Fig. 3G). Among the 31 genera analyzed, *Faecalibaculum*, *Lactobacillus*, *Ligilactobacillus* were significantly reduced, whereas *norank_o_Clostridia_UCG-014*, *Bacteroides*, *Parabacteroides*, and *Prevotellaceae_UcG-001* were expanded in PU mice (Fig. 3H). LEfSe/LDA corroborated these signatures (LDA > 4; Fig. 3I-J), confirming that specific bacterial taxa are key drivers of intestinal dysbiosis.

**Fig. 4:**
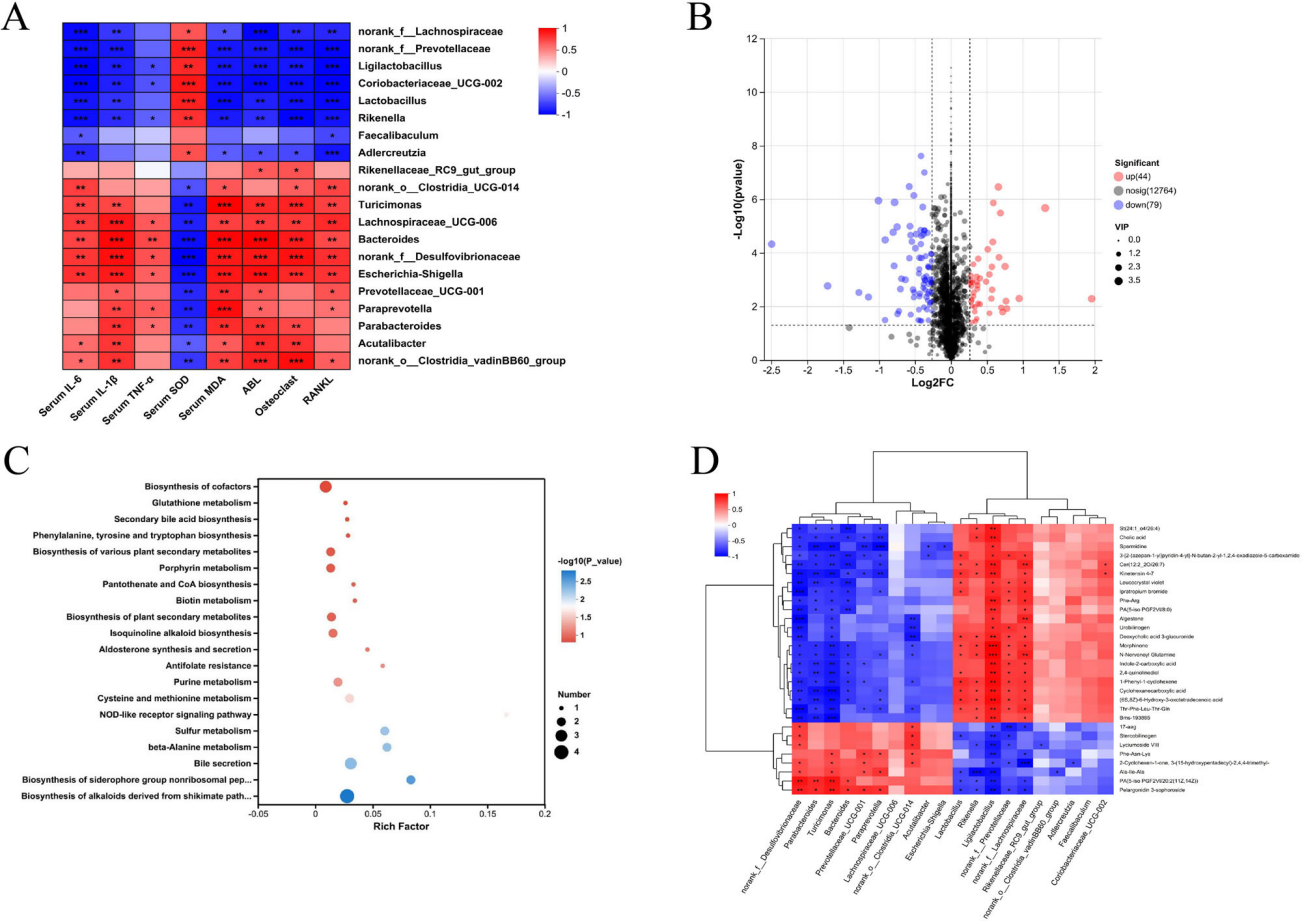
UC drives metabolomic rewriting in periodontitis mice (*N* = 5/group). **A** Heatmap of Spearman correlation coefficients between the top 20 differential gut bacterial genera affected by UC and periodontitis-related parameters. **B** Volcano plot illustrating 123 differentially accumulated metabolites, with log2(FoldChange) represented on the *x*-axis and −log10 (*p*-value) on the *y*-axis, highlighting significant alterations between the P and PU groups. **C** Kyoto Encyclopedia of Genes and Genomes (KEGG) pathway enrichment scatter plot shows the changes in metabolic pathways of fecal metabolites in periodontitis mice affected by UC. **D** Pearson correlation coefficients between the top 20 differentially enriched gut bacterial genera and the top 30 differential metabolites, with correlation effects indicated by a color gradient ranging from red (positive correlation) to blue (negative correlation).

Untargeted LC–MS metabolomics of feces identified 123 differentially abundant metabolites (44 upregulation, 79 downregulation in periodontitis mice; Fig. 4B). KEGG mapping indicated that glutathione metabolism, secondary bile acid biosynthesis, and the biosynthesis pathways for phenylalanine, tyrosine, and tryptophan were the most perturbed (Fig. 4C). Spearman correlation integrating the top 20 discriminant genera of intestinal flora with eight host physiological indicators (alveolar bone resorption, inflammatory factors, and oxidative stress markers) revealed that *norank_o_Clostridia_UCG-014*, *Turicimonas*, *Bacteroides*, *norank_f_Desulfovibrionaceae*, *Escherichia-Shigella*, *Prevotellaceae_UCG-001*, *Paraprevotella*, *Parabacteroides*, and *Acutalibacter* positively correlated with bone metabolism, MDA, and pro-inflammatory cytokines (Fig. 4A), but negatively correlated with amino acids and bile acid biosynthetic modules (Fig. 4D).

Predictive functional profiling based on 16S sequencing data (PICRUSt2) assigned the functional profiles of the PU group to the KEGG pathways enriched in metabolic pathways, biosynthesis of secondary metabolites, microbial metabolism in diverse environments, biosynthesis of amino acids, carbon metabolism, ribosome biogenesis, ABC transporters, purine metabolism, two-component systems, and amino acid and ribosomal metabolism (Figure S1C), supporting the metabolomic evidence of an altered metabolic landscape.

Collectively, these data demonstrate that UC imposes a dysbiotic gut microbiota with concomitant metabolomic shifts that associate with heightened systemic inflammation and periodontal bone destruction.

### Causal impact of dysbiotic gut microbiota on periodontitis

#### FMT-derived dysbiosis intensifies intestinal inflammation, barrier failure, and mesenteric Th17/Treg imbalance

**Fig. 5:**
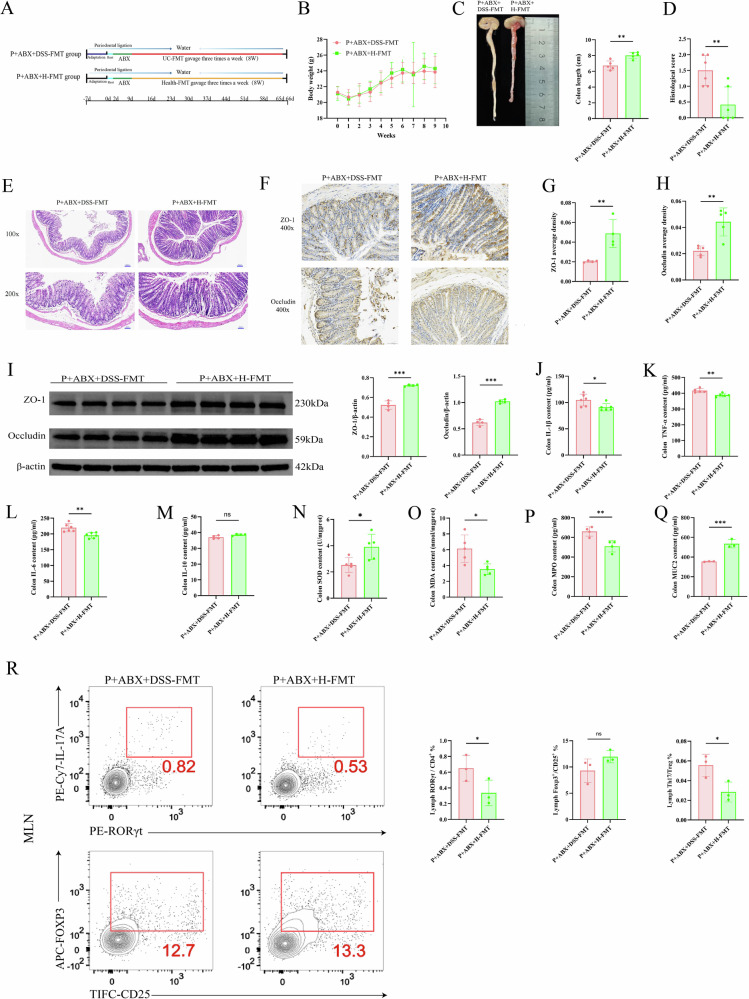
FMT-derived dysbiosis intensifies intestinal inflammation, barrier failure, and mesenteric Th17/Treg imbalance. **A** Experimental design: Germ-restricted mice pretreated with broad-spectrum antibiotics for 7 days, followed by periodontitis induction and FMT from DSS-UC or healthy mice (three times a week for 8 consecutive weeks). **B** Body weight changes (*N* = 8/group). **C** Colon length (*N* = 5/group). **D**, **E** Representative images of hematoxylin and eosin (H&E)-stained colon tissues (100× and 200× magnification, Scale bar: 100 μm and 50 μm, *N* = 6/group). **F**–**H** Representative immunohistochemical images and quantification of occludin (*N* = 5/group) and zonula occludens-1 (ZO-1, *N* = 4/group) in the distal colon (400× magnification, Scale bar: 50 μm). **I** Western blot analysis of Occludin and ZO-1 relative to β-actin (*N* = 4/group). **J**–**M** Intestinal inflammation levels were assessed by measuring the concentrations of pro-inflammatory cytokines IL-1β, IL-6, and TNF-α, as well as the anti-inflammatory cytokine IL-10 (*N* = 4-6/group). **N**, **O** Oxidative stress status was evaluated by measuring the SOD and MDA levels (*N* = 5/group). **P**, **Q** Intestinal myeloperoxidase (MPO, *N* = 4/group) and mucin 2 (MUC2, *N* = 3/group) levels. **R** Proportions of Th17 (CD4⁺RORγt⁺IL-17A⁺) and Treg (CD4⁺CD25⁺Foxp3⁺) cells (% of CD4^+^ gate) and Th17/Treg ratio in mesenteric lymph nodes (*N* = 3/group). Data are expressed as mean ± standard error of the mean (SEM). *P < 0.05; **P < 0.01; ***P < 0.001.

To determine whether colitis-associated microbiota is sufficient to drive periodontal bone loss, we transplanted fecal microbiota from DSS-colitic or healthy donors into antibiotic-pretreated, ligature-induced periodontitis mice (P + ABX + DSS-FMT vs P + ABX + H-FMT). Recipients of the dysbiotic microbiota exhibited overt colitis: shorter colon length (Fig. 5C), higher colonic histopathological scores (Fig. 5D-E), elevated mucosal IL-6, IL-1β, TNF-α, and MPO activity (Fig. 5J-L, P), together with decreased SOD activity and increased MDA levels (Fig. 5N, O). Tight-junction proteins Occludin and ZO-1 were markedly downregulated (Fig. 5F-I, Figure S3) and goblet-cell mucin 2 (MUC2) decreased (Fig. 5Q), indicating severe barrier damage.

Flow cytometry of mesenteric lymph nodes (MLNs) revealed a significant rise in RORγt⁺CD4⁺ T cells, and the Th17/Treg ratio in the P + ABX + DSS-FMT group (Fig. 5R), demonstrating that transfer of dysbiotic gut flora is sufficient to break mucosal immune homeostasis and amplify local inflammation in the context of periodontitis.

#### Dysbiotic gut microbiota drive systemic inflammation, splenic Th17/Treg skewing and alveolar bone resorption

**Fig. 6:**
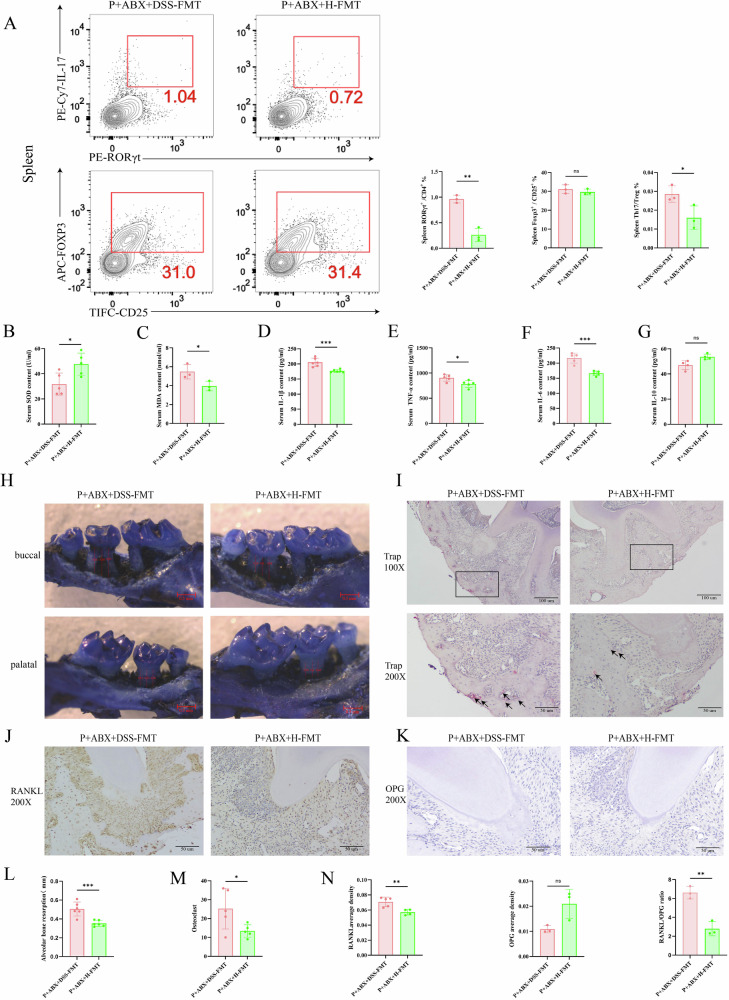
Dysbiotic gut microbiota drive systemic inflammation, splenic Th17/Treg skewing, and alveolar bone resorption. **A** Proportions of Th17 (CD4⁺RORγt⁺IL-17A⁺) and Treg (CD4⁺CD25⁺Foxp3⁺) cells within the CD4^+^ gate, and Th17/Treg ratio in the spleen (*N* = 3/group). **B**, **C** Serum oxidative stress markers (SOD, *N* = 5/group and MDA, *N* = 3/group). **D**–**G** Serum cytokine levels (IL-1β, IL-6, TNF-α, and IL-10; *N* = 5–6/group). **H**, **L** Stereomicroscopic images and quantitative analysis of alveolar bone resorption (*N* = 6/group, Scale bar: 0.5 mm). **I**, **M** Representative TRAP staining images and quantification of multinucleated osteoclasts in periodontal tissues below the furcation and near the root apex (100× and 200× magnification, *N* = 5/group, Scale bar: 100 μm and 50 μm). **J**, **N** Representative immunohistochemical images and quantification of RANKL in alveolar bone adjacent to the root surface (*N* = 4/group, Scale bar: 50 μm). **K**, **N** Representative immunohistochemical images and quantification of OPG and RANKL/OPG ratio in alveolar bone adjacent to the root surface (*N* = 3/group, Scale bar: 50 μm). Data are expressed as mean ± standard error of the mean (SEM). *P < 0.05; **P < 0.01; ***P < 0.001.

The FMT further propagated inflammation beyond the gut. Splenic Th17 frequency and the Th17/Treg ratio were both higher in P + ABX + DSS-FMT mice than in healthy-microbiota controls (Fig. 6A). Circulating IL-6, IL-1β, and TNF-α increased in parallel, while the anti-inflammatory factor IL-10 remained unchanged; oxidative-stress markers followed the same trend observed in the colon (Fig. 6B-G).

Stereomicroscopic imaging and measurement of maxillae showed significantly greater alveolar-crest resorption (Fig. 6H-L, Figure S2A). Consistent with the comorbidity model, TRAP staining and RANKL immunohistochemical detection jointly demonstrated that the number of osteoclasts in periodontal tissues significantly increased in the P + ABX + DSS-FMT group compared to the healthy donor, and the expression level of RANKL protein was markedly upregulated, whereas OPG expression decreased without statistical significance, leading to a markedly increased RANKL/OPG ratio (Figs. 6I-K, 6M-N).

Collectively, these findings suggest that dysbiotic gut microbiota act as a transmissible factor that systemically biases Th17 immunity and accelerates osteoclastic bone loss.

#### Dysbiotic gut microbiota restructure the oral microecosystem

**Fig. 7:**
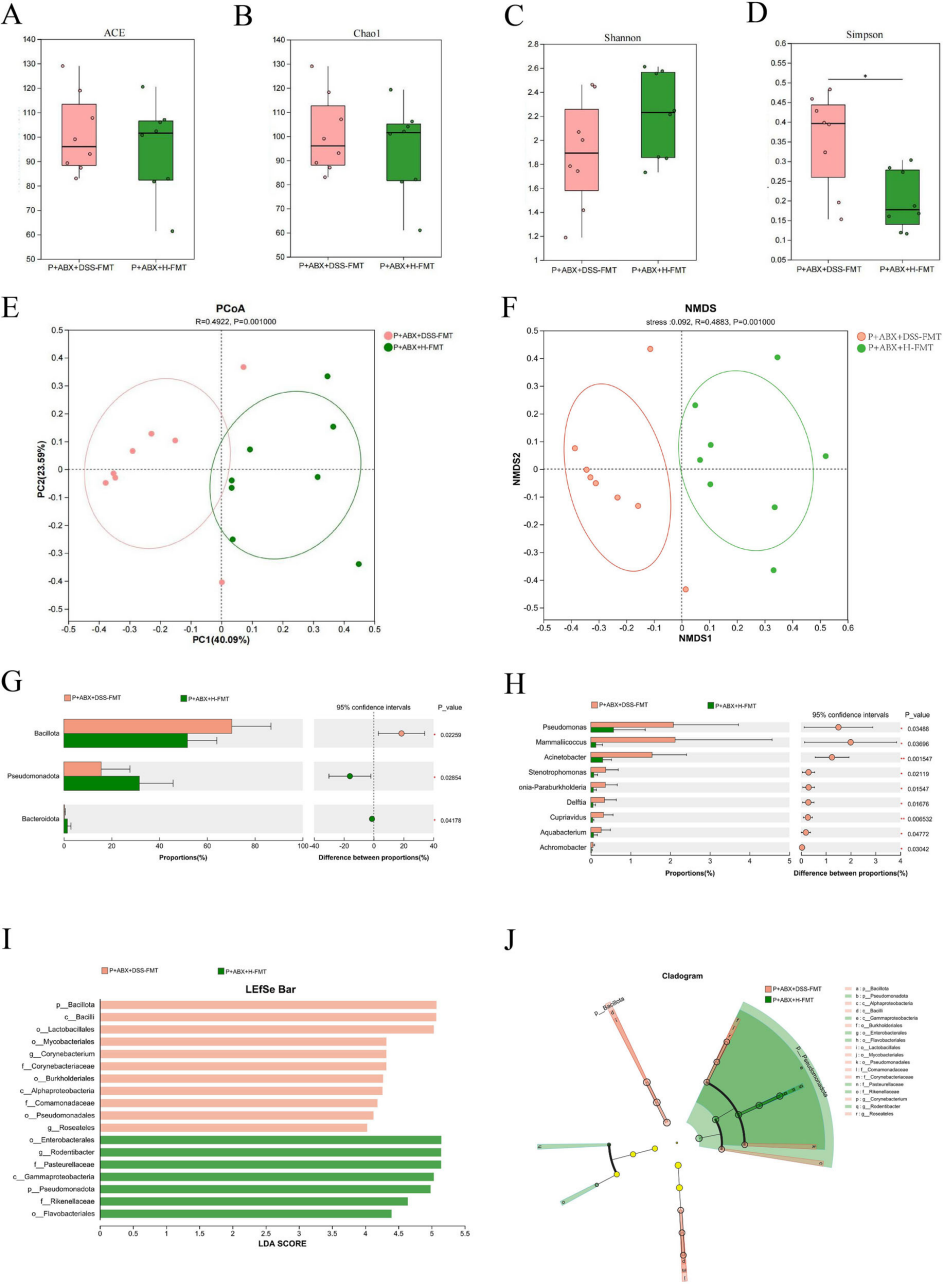
Dysbiotic gut microbiota restructure the oral microecosystem (*N* = 8/group). **A**–**D** Alpha diversity indices (ACE, Chao1, Shannon, and Simpson) in the P + ABX + DSS-FMT and P + ABX + H-FMT groups. **E** Oral microbiota profiles were examined by PCoA based on Bray–Curtis distance. **F** NMDS analysis (*R* > 0, indicating that inter-group differences exceed intra-group variation). **G**, **H** Relative abundance of bacteria at the phylum (**G**) and genus (**H**) levels. **I** LEfSe analysis showing biomarkers (LDA > 4). **J** Cladogram of LEfSe multi-level species discriminant analysis (LDA > 4).

Saliva 16S rRNA profiling was performed 8 weeks post-FMT. Alpha-diversity indices did not differ between P + ABX + DSS-FMT and P + ABX + H-FMT groups (Fig. 7A-D), yet PCoA and NMDS analysis revealed a distinct beta-diversity cluster (Fig. 7E-F), indicating successful oral microbial reconfiguration.

Student’s t-test identified significant alterations in three bacterial phyla, including *Bacillota*, and nine genera such as *Pseudomonas* (Fig. 7G-H). LEfSe (LDA > 4) nominated *Bacillota*, *Corynebacterium* and *Roseateles* as the most discriminatory taxa in P + ABX + DSS-FMT group (Fig. 7I-J).

PICRUSt2 inference indicated enrichment of pathways related to metabolic pathways, biosynthesis of secondary metabolites, microbial metabolism in diverse environments, amino acids biosynthesis, ABC transporters, carbon metabolism, ribosome biogenesis, purine metabolism, two-component systems, and quorum sensing (Figure S2B), implying a functional shift toward nutrient scavenging and inter-species signaling.

#### Dysbiotic consortium persistently alters intestinal microbiota and depletes colonic SCFAs

**Fig. 8:**
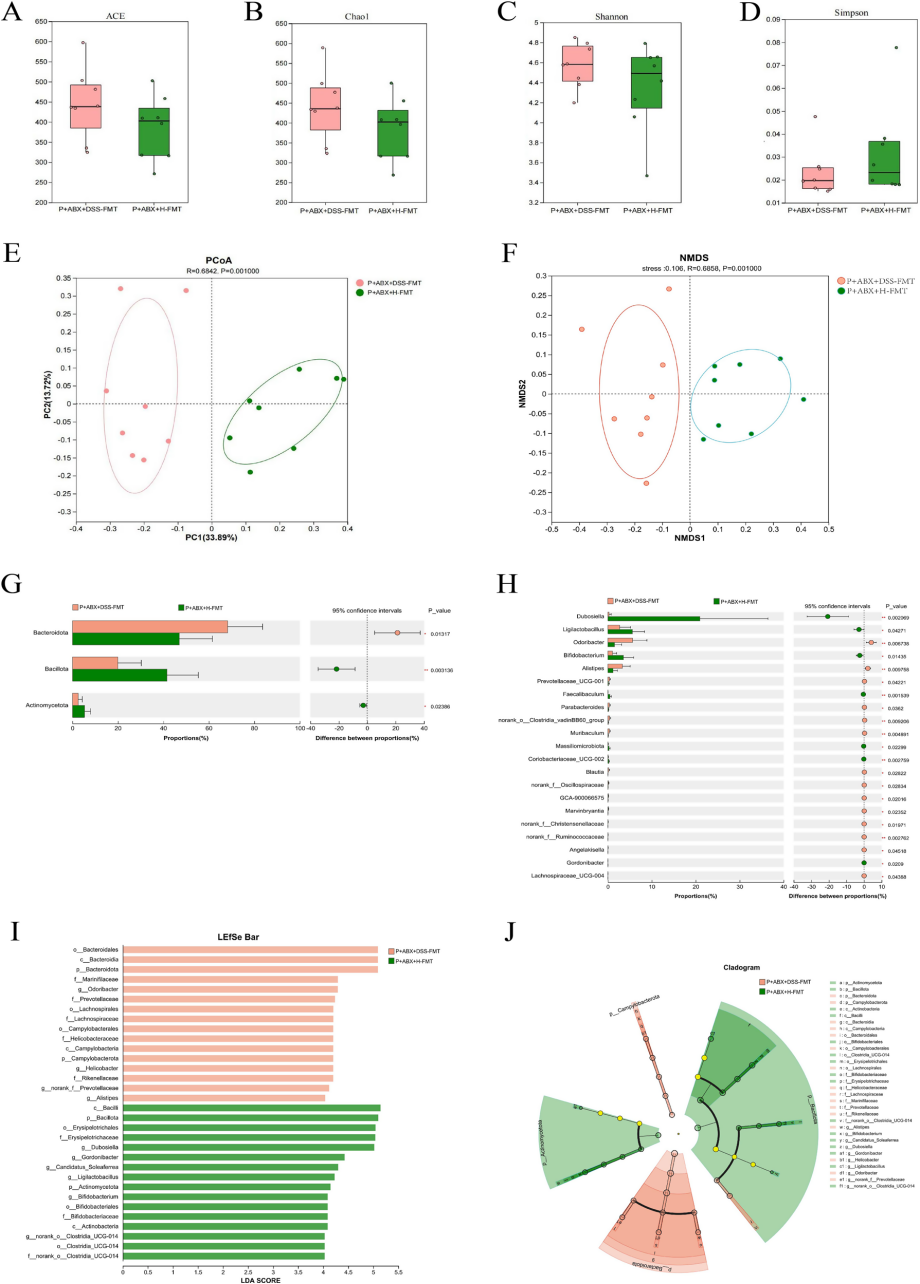
Dysbiotic consortium persistently alters intestinal microbiota (*N* = 8/group). **A**–**D** Alpha diversity indices (ACE, Chao1, Shannon, and Simpson) in the P + ABX + DSS-FMT and P + ABX + H-FMT groups. **E** Gut microbiota profiles were examined by PCoA based on Bray–Curtis distance. **F** NMDS analysis (*R* > 0, indicating that inter-group differences exceed intra-group variation). **G**, **H** Relative abundance of bacteria at the phylum (**G**) and genus (**H**) levels in the P + ABX + DSS-FMT and P + ABX + H-FMT groups. **I** LEfSe analysis revealing biomarkers (LDA > 4). **J** Cladogram of LEfSe multi-level species discriminant analysis (LDA > 4).

Fecal 16S rRNA sequencing confirmed stable engraftment: alpha-diversity remained equivalent (Fig. 8A-D), whereas PCoA and NMDS showed complete segregation of P + ABX + DSS-FMT and P + ABX + H-FMT samples (Fig. 8E-F).

Compared to healthy-microbiota controls, Bacteroidota expanded at the expense of *Bacillota*, and *Actinomycetota*; 21 genera—headed by *Alistipes*, *Prevotellaceae_UCG-001*, *Parabacteroides*, and *Muribaculum*—were significantly altered in P + ABX + DSS-FMT mice (Fig. 8G-H). LEfSe highlighted these taxa as signature biomarkers (Fig. 8I-J). Notably, a total of nine differential microbial taxa — including eight genera and one phylum — overlapped with those that distinguish DSS-colitis mice in the original comorbidity model, highlighting a conserved dysbiosis signature.

**Fig. 9:**
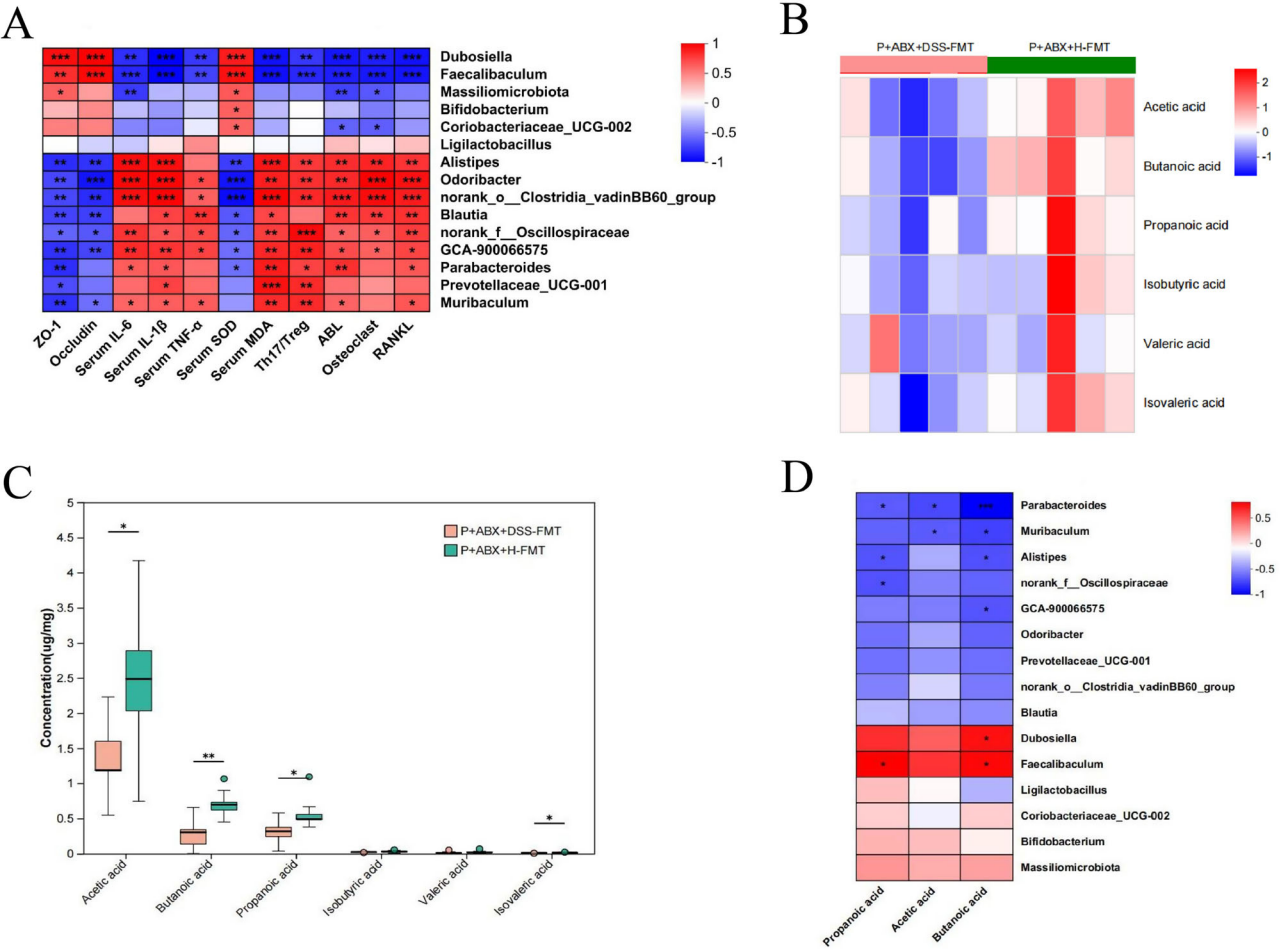
Dysbiotic consortium depletes colonic SCFAs (*N* = 5/group). **A** Spearman correlation heatmap between the top 15 gut bacterial genera and parameters related to periodontitis, intestinal barrier function, and Th17/Treg balance. **B** Cluster analysis heatmap of the analyzed parameters. **C** Changes in short-chain fatty acids (SCFAs) content, including acetic acid, butanoic acid, propanoic acid, isobutyric acid, valeric acid, and isovaleric acid. **D** Spearman correlation heatmap between the top 10 gut bacterial genera and SCFAs (acetic acid, butanoic acid, and propanoic acid). Data are expressed as mean ± standard error of the mean (SEM). *P < 0.05; **P < 0.01; ***P < 0.001.

Targeted GC–MS quantification of colonic contents revealed a near-complete divergence in SCFA profiles: acetate, propionate, butyrate, and isovalerate were all significantly lower by 35–60% in the P + ABX + DSS-FMT group (Fig. 9B, C). Spearman integration with host parameters showed that *Alistipes*, *Odoribacter*, *norank_o_Clostridia_vadinBB60_group*, *Blautia, norank_f_Oscillospiraceae*, *GCA-900066575, Parabacteroides*, *Prevotellaceae_UCG-001*, and *Muribaculum* positively correlated with bone metabolism, Circulating MDA, pro-inflammatory cytokines, and spleen Th17/Treg imbalance (Fig. 9A), but negatively with SCFA concentrations (Fig. 9D), implicating these taxa as keystone organisms linking metabolite depletion to inflammatory bone destruction.

Furthermore, PICRUSt2-predicted functional profiles (KEGG level-3) showed enrichment of microbial amino-acid and carbohydrate metabolism, ABC transporters, and quorum-sensing pathways in the P + ABX + DSS-FMT group (Figure S2C), consistent with the metabolomics-derived shortage of SCFAs and enhanced proteolytic environment.

#### Dysbiotic microbiota reprogram colonic gene expression

**Fig. 10:**
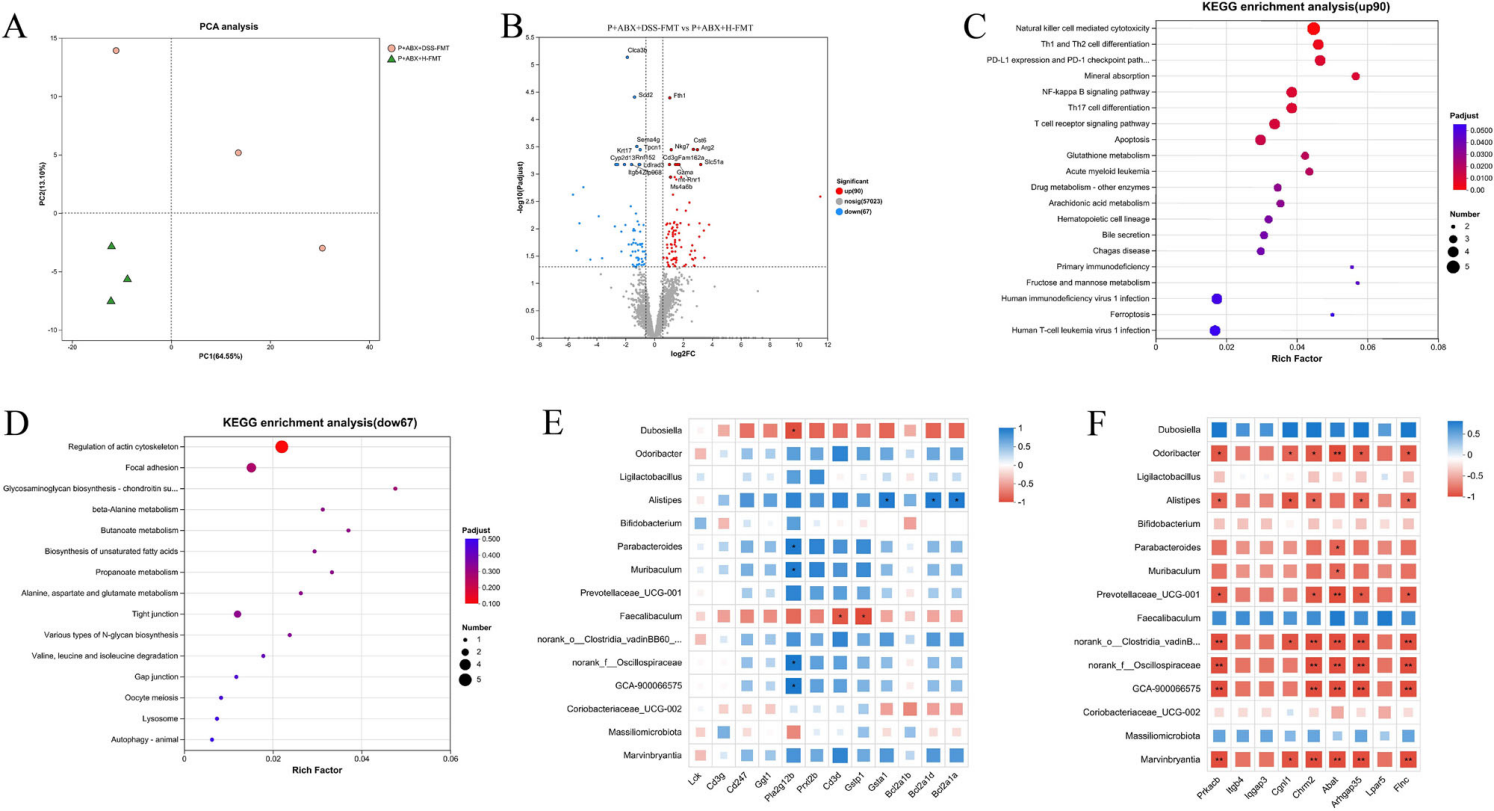
Dysbiotic microbiota reprogram colonic gene expression (*N* = 3/group). **A** Principal component analysis (PCA) plot of colonic transcriptomes from the P + ABX + DSS-FMT (pink) and P + ABX + H-FMT (green) groups. **B** Volcano plots depicting 157 differentially expressed genes (DEGs) with fold change ≥ 1.5. **C**, **D** KEGG functional enrichment analysis of 90 upregulated (**C**) and 67 upregulated (**D**) DEGs. **E** Correlations between gut microbiota and functional genes involved in immune response, lipid metabolism, or amino acid-related pathways enriched in upregulated DEGs. **F** Correlation between gut microbiota and functional genes associated with actin cytoskeleton regulation, intestinal barrier integrity, and SCFAs metabolism enriched in downregulated DEGs.

Colon RNA -Seq was performed on FMT recipients. Principal component analysis (PCA) plots demonstrated clear transcriptomic separation (Fig. 10A). Volcano filtering (|Fold change | ≥1.5, adjusted *P*-value < 0.05) yielded 90 upregulated and 67 downregulated genes in P + ABX + DSS-FMT mice (Fig. 10B). KEGG functional clustering revealed enrichment of immune response, amino acid metabolism, and lipid metabolism among upregulated genes (Fig. 10C), whereas epithelial barrier function and SCFA metabolic pathways dominated the downregulated set (Fig. 10D). qRT-PCR validated eight representative differentially expressed genes (DEGs; *CD3g*, *CD247*, *Cd3d*, *Bc2alb* increased; *Itgb4*, *Arngap35*, *Prkacb*, *Cgnl1* decreased) in the same direction as RNA-seq (Fig. S2D).

Spearman correlation between colonic bacterial genera and selected host DEGs (12 immunity- or lipid-/amino-acid-metabolism-related upregulated genes and 9 epithelial-barrier or SCFA-metabolism-related downregulated genes) disclosed that *Parabacteroides* and *Muribaculum* positively associated with the lipid-metabolism genes *Pla2g12b* but negatively with SCFA-metabolism related genes *Abat*; *Alistipes* correlated positively with inflammatory genes (*Bc2ald*, *Bcl2ala*) and amino-acid metabolism (*Gstal*), while negatively with epithelial-barrier related transcripts (*Chrm2*, *Arhgap35*, *Prkacb*, *Cgnl1*, and *Finc*) (Fig. 10E, F). These data mechanistically link expansions of specific gut-resident bacteria to impaired SCFA metabolism, compromised barrier integrity, and heightened mucosal immunity.

### Periodontitis reciprocally exacerbates UC

**Fig. 11:**
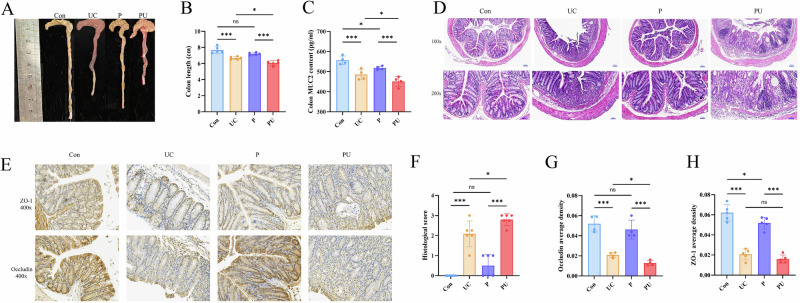
Periodontitis reciprocally exacerbates UC. **A**, **B** Colon length in the Con, UC, P, and PU groups (*N* = 5/group). **C** Intestinal MUC2 levels (*N* = 4/group). **D**, **F** Representative H&E-stained colon tissue images (100× and 200× magnification, *N* = 6/group, Scale bar: 100 μm and 50 μm). **E**, **G**, **H** Representative immunohistochemical images and quantification of Occludin (*N* = 4/group) and ZO-1 (*N* = 5/group) in the distal colon (400× magnification, Scale bar: 50 μm). Data are expressed as mean ± standard error of the mean (SEM). *P < 0.05; **P < 0.01; ***P < 0.001.

Finally, we examined the reverse direction of the gut–oral axis. Compared with UC alone, PU mice exhibited more severe colitis: markedly shortened colon length (Fig. 11A, B), reduced MUC2 (Fig. 11C), extensive goblet-cell loss and inflammatory infiltration on HE staining (Fig. 11D, F), together with decreased tight-junction proteins Occludin and ZO-1 (Fig. 11E, G, H). Thus, periodontitis can act as a systemic inflammatory amplifier that further compromises intestinal barrier function and perpetuates the inflammatory loop between the oral cavity and intestines.

## Discussion

The oral cavity and intestine form a continuous mucosal interface that harbors the two densest microbial ecosystems of the human body. While periodontitis has traditionally been viewed as a site-specific dysbiosis driven by oral pathobionts and an exaggerated local immune response^24^. A growing body of evidence now implicates the gut microbiota in the systemic regulation of bone metabolism and, consequently, periodontal bone remodeling^25,26^. Conversely, periodontal bacteria and their inflammatory mediators can reach the gut and exacerbate enteritis^11,27^. The present study provides the first experimental demonstration that the relationship is bidirectional: DSS-induced UC not only reconfigures the oral microbiota but also accelerates ligature-induced alveolar bone loss, and this process is transmissible by FMT.

Mice with concurrent UC and periodontitis exhibited pronounced cachexia, splenomegaly, elevated serum IL-6, IL-1β, and TNF-α, and oxidative stress (decreased SOD and increased MDA), mirroring the serological profile reported in IBD patients with comorbid periodontitis^28,29^. These systemic alterations coincided with increased RANKL/OPG ratio and TRAP^+^ osteoclast numbers, resulting in marked crestal bone resorption. Notably, the increased RANKL/OPG ratio observed in both the comorbidity model and FMT recipients suggests that UC-driven gut dysbiosis promotes a pro-osteoclastogenic environment primarily through RANKL upregulation rather than OPG downregulation, indicating a pro-osteoclastogenic environment. Although OPG expression showed a decreasing trend, the lack of statistical significance indicates that RANKL elevation is likely the dominant driver of periodontal bone loss in this model. Although we did not directly assess osteoblast-specific markers, the presence of net bone loss despite enhanced osteoclast activity, together with the relative reduction in OPG—which is partly produced by osteoblasts and stromal cells—is consistent with a potential impairment of osteoblastic compensation^30,31^. In addition, the unchanged IL-10 levels may indicate a lack of compensatory anti-inflammatory response, further contributing to exacerbated inflammation in the comorbid state. Future studies incorporating dynamic histomorphometry, such as calcein labeling, will be necessary to directly quantify osteoblast function and bone formation rates.

16S profiling of saliva revealed that UC selectively enriched intestinal pathobionts—*Escherichia–Shigella Coriobacteriaceae UCG-002*, and *Morganella*—in the oral cavity. Additionally, differential changes in genera such as *Corynebacterium* and *Roseateles* were observed in the oral cavity of periodontitis mice receiving UC-derived dysbiotic microbiota via FMT. The presence of these intestinal pathobionts in the oral cavity argues against transient spill-over and suggests ectopic colonization, probably via hematogenous or lymphatic routes. The hematogenous dissemination of oral pathogens such as *F. nucleatum* has been well-documented in IBD and colorectal cancer^32^. Notably, recent evidence indicates that *F. nucleatum* can survive within neutrophils and be disseminated to distant sites via a “Trojan horse” mechanism, in which immune cells act as carriers for microbial transport^33^. PICRUSt2 analysis further showed that UC-associated oral microbiota remodeling was accompanied by the enrichment of functional pathways like amino acid biosynthesis and quorum sensing. These ectopically colonized intestinal pathobionts and FMT-induced differential oral genera may synergize with resident oral periodontal pathogens through metabolic cooperation and inter-species signaling to exacerbate local inflammation and tissue destruction^27^. Furthermore, FMT experiments demonstrated that transplantation of dysbiotic gut microbiota from UC mice reshaped the recipient oral microecology, including phylum-level changes in *Bacillota*, supporting a gut-to-oral microbial translocation.

It should be noted that, due to primer specificity and limited taxonomic resolution of 16S rRNA sequencing, key periodontal pathogens—including *P. gingivalis*, *Tannerella forsythia*, *Treponema denticola*, and emerging pathogens such as *Filifactor alocis*—were not reliably detected in this study. Future shotgun metagenomic or species-specific qPCR will be necessary to more precisely evaluate the role of specific pathogens in the gut–oral axis.

In the UC-periodontitis comorbidity model, fecal analysis identified a core UC-associated signature characterized by expansion of Bacteroidetes (including *Bacteroides*, *Parabacteroides*, and *Prevotellaceae_UCG-001*) and contraction of Firmicutes (including *Ligilactobacillus* and *Faecalibaculum*)—a pattern that recapitulates the dysbiotic profile observed in human IBD^34,35^. The same nine discriminatory gut microbial taxa — including eight genera and one phylum—were detected in both the original DSS-UC comorbidity model (PU vs. P) and in subsequent FMT recipient mice (P + ABX + DSS-FMT vs. P + ABX + H-FMT), demonstrating that this microbial signature is reproducible across individuals and portable via FMT.

Targeted metabolomics showed that a reduction of 35–60% in colonic acetate, propionate, and butyrate in FMT recipients of UC mouse microbiota, paralleling the decreased fecal SCFA concentrations reported in IBD patients^36,37^. Correlation analysis indicated that the abundance of nine intestinal genera, including *Parabacteroides*, *Muribaculum*, and *Alistipes*, was negatively correlated with SCFA levels, positively correlated with the Th17/Treg ratio, and further associated with alveolar bone loss (Fig. 9A, D). These findings align with an in vitro study demonstrating that butyrate suppresses osteoclastogenesis via histone deacetylase (HDAC) inhibition and GPR43 activation^21^. The expansion of these Bacteroidetes taxa (like *Parabacteroides* and *Muribaculum*) does not directly deplete SCFAs but competitively displaces butyrate-producing Firmicutes, leading to metabolic shortage of bone-protective SCFAs^38^, tilting Th17/Treg imbalance toward inflammation and exacerbating osteoclastic bone resorption^39^.

RNA-seq of colon tissues from FMT recipient mice revealed that UC-associated dysbiotic microbiota induced the upregulation of 90 genes and downregulation of 67 genes: the upregulated genes were mainly enriched in pathways related to immune response (especially Th17 cell activation; such as *CD3g*, *CD247*, and *Cd3d*), amino acid metabolism (e.g., *Abat*, *Gstal*), and lipid metabolism, while the downregulated genes were concentrated in epithelial barrier function (such as *Itgb4*, *Arhgap35*, and *Cgnl1*) and SCFA metabolism pathways. Correlation analysis linked *Parabacteroides* and *Muribaculum* to reduced *Abat* expression—*Abat* is a mitochondrial enzyme that sustains TCA-cycle flux and nucleotide salvage^40^, has been identified as a downstream target of Dnmt3b with potential as a therapeutic target for osteoarthritis^41^.

Knockdown of *Abat* can activate type I interferon (IFN-I) and IFN-γ-related signaling pathways while inhibiting secondary metabolism and arginine metabolism processes^42^. Their expansion may thus deplete epithelial ATP and mtDNA, compromise tight-junction integrity, and tilt the Th17/Treg balance toward inflammation by reducing succinate-driven HIF-1α/STAT3 signaling^42^. *Alistipes* was positively correlated with inflammation-related genes (*Bc2ald*, *Bcl2ala*) and amino acid metabolism gene *Gstal* that promote Th17 cell differentiation, and negatively correlated with epithelial barrier genes (*Chrm2*, *Arhgap35*, etc.) (Fig. 10E, F). Collectively, the data indicate that UC-associated microbiota epigenetically prime the colonic mucosa for a pro-inflammatory, pro-osteoclastic systemic milieu.

Immune dysregulation, particularly Th17/Treg imbalance, links gut dysbiosis to inflammatory bone loss^43^. We observed that transplantation of UC-derived dysbiotic microbiota significantly raised the frequency of IL-17⁺CD4⁺ T cells and the Th17/Treg ratio in mesenteric lymph nodes and spleens of recipient mice (Fig. 5R, Fig. 6A), which was closely associated with alveolar crest resorption (Fig. 9A). Correlation analysis linked the key dysbiotic genera (e.g., *Parabacteroides*, *Muribaculum*, *Alistipes*) to this skewing, indicating these taxa are potential upstream drivers of Th17/Treg imbalance.

Two parallel Mechanisms appear to operate: first, depletion of SCFAs reduces the inhibitory effect on Th17 cell differentiation while weakening colonic barrier integrity; second, upregulation of Th17-signature genes (*CD3g*, *CD247*, etc.) and downregulation of amino acid metabolism-related gene (*Abat*) indirectly promote Th17 cell expansion and inhibit Treg cell function^44–46^. This Th17/Treg imbalance not only enhances systemic inflammation but also directly promotes osteoclastic activation by driving RANKL expression^47^, ultimately exacerbating inflammatory alveolar bone loss in periodontitis^48^.

A limitation of this study is the lack of direct Th17/Treg analysis in oral tissues. Due to technical constraints (minute tissue volume and low cell yield), we analyzed these cells in the spleen and MLNs as surrogate markers of systemic immune status. Future studies employing single-cell RNA sequencing may enable characterization of oral tissue-resident T cell subsets.

Bidirectional translocation of oral-gut pathobionts is one of the core mechanisms linking the two diseases, and this process is closely associated with Th17/Treg imbalance. Recent studies have confirmed that oral *P. gingivalis* can translocate to the intestine and exacerbate DSS-induced colitis by inhibiting linoleic acid-aryl hydrocarbon receptor (AHR) signaling, thereby tilting the Th17/Treg balance toward mucosal inflammation^11,27^. Our results further provide supporting evidence: periodontitis can significantly exacerbate UC symptoms, manifested by shortened colon length, depleted MUC2, loss of goblet cells, and decreased tight junction proteins (Occludin/ZO-1) expression (Fig. 11), which may be mediated by oral-derived pathobionts translocating to the intestine and inducing Th17/Treg imbalance. Meanwhile, our study reveals the mechanistic mirror image of this axis: UC-derived dysbiotic microbiota (enriched in *Parabacteroides*, *Muribaculum*, *Alistipes*, etc.) travel in the opposite direction to reshape the salivary microenvironment (inducing the enrichment of differential genera such as *Corynebacterium*), deplete SCFAs, and systemically expand Th17 cells, ultimately accelerating osteoclastic alveolar bone resorption (Figs. 5S and 6–9). These data define periodontitis and colitis as inter-mucosal inflammatory syndromes linked by trans-compartmental pathobiont translocation, microbiota-gene interactions, and metabolite-driven Th17/Treg skewing, supporting an integrated oral-systemic management strategy in patients with either disease.

PU Mice displayed more severe colitis than those in the UC-only controls: shortened colon length, depleted MUC2, loss of goblet cells, decreased expression of Occludin/ZO-1, and increased mucosal inflammatory infiltration. This reverse axis is consistent with reports that oral pathogens translocate to the gut and amplify DSS-induced colitis^49,50^, and underscores the bidirectional gut–oral inflammatory loop as a pathophysiological entity.

This study confirmed that UC can exacerbate periodontitis bone resorption; more importantly, UC-related intestinal flora imbalance through metabolites and Th17-mediated pathways is both necessary and sufficient to aggravate periodontitis. These findings indicate that restoring SCFA-producing Firmicutes or direct SCFA supplementation may represent a feasible adjuvant therapy for periodontal patients with comorbid IBD^21,51^. Probiotics and prebiotics have shown efficacy in UC through microbiota regulation, barrier enhancement, and immune modulation^52–54^. Given shared pathogenic mechanisms (dysbiosis, Th17/Treg imbalance), such interventions could address both conditions simultaneously. However, high-quality randomized controlled trial evidence supporting this cross-disease effect is currently lacking and warrants future investigation. Further work should identify optimal probiotic strains, dosing regimens, and treatment windows through long-read sequencing and metabolomics of paired fecal and salivary samples, followed by interventional trials testing encapsulated SCFAs or next-generation Firmicutes-based probiotics.

## Methods

### Animals

Seven-week-old male C57BL/6 J mice, weighing approximately 18 ± 2 g, were purchased from Hunan Slick-Jinda Laboratory Animal Co., Ltd. (License No.: SCXK (Xiang) 2021-0002) and housed in the specific pathogen-free (SPF) Experimental Animal Center of Zunyi Medical University under controlled environmental conditions (temperature: 20–24 °C; humidity: 45–60%; 12-h light/dark cycle). All experimental procedures and animal manipulations were approved by the Ethics Committee of Zunyi Medical University on January 26, 2021 (Approval No.: ZYLS [2021]1-046) and conducted in strict accordance with the ARRIVE guidelines.

After 7 days of acclimatization, the mice were randomly divided into four groups: Control (Con), Ulcerative Colitis (UC), Periodontitis (P), and Periodontitis combined with Ulcerative Colitis (PU). Under general anesthesia (1% pentobarbital sodium, 100 μl/20 g body weight, intraperitoneal injection), mice in the P and PU groups received bilateral maxillary second-molar ligation using 5-0 silk suture. Ligatures were inspected every 48 h and replaced if detached until euthanasia. Following a 2-day recovery period, UC and PU mice were given 2.0% (w/v) dextran sulfate sodium (DSS; molecular weight: 36,000–50,000 Da; MP Biomedicals, Cat. #160110) in drinking water for 5 consecutive days, followed by distilled water for 10 days; this cycle was repeated 2.5 times. Fresh fecal samples were collected from Con and UC mice during the active phase of colitis for subsequent FMT assays.

A separate cohort of mice was used for FMT studies, which were assigned to two groups: Periodontitis + Antibiotic Pretreatment + Colitis Mouse of Fecal Microbiota Transplantation (P + ABX + DSS-FMT) and Periodontitis + Antibiotic Pretreatment + Healthy Mouse Fecal Microbiota Transplantation (P + ABX + H-FMT). Periodontitis was induced as previously described. After a 2-day recovery period, mice were administered an antibiotic cocktail (1 g/l ampicillin, 1 g/l neomycin, 1 g/l metronidazole, and 0.5 g/l vancomycin; Aladdin Reagent (Shanghai) Co., Ltd.) in drinking water for 1 week to establish a germ-reduced mouse model. The fecal suspension was freshly prepared prior to each gavage: 100 mg of fecal pellets from either UC or Con donors were suspended in 1 ml sterile 0.9% physiological saline, vortexed thoroughly, and centrifuged (5000 rpm, 4 °C, 15 min). The supernatant (10 μl/g) was administered by oral gavage three times per week for 8 weeks.

### Samples collection and tissue processing

Throughout the experimental period, mice had ad libitum access to standard chow and water; body weight was recorded daily. Upon completion of the experiment, after a 12-h fast, fresh fecal and saliva were collected, snap-frozen in liquid nitrogen, and stored at −80 °C. Animals were then deeply anesthetized with 1% pentobarbital sodium (i.p. 50 mg kg^−^¹). Blood samples were obtained from the retro-orbital plexus into serum-separator tubes, kept on ice for 30 min, and centrifuged (3000 rpm, 4 °C, 10 min); serum was aliquoted and stored at −80 °C^26,55^. Spleens and mesenteric lymph nodes were excised, weighed, and placed in ice-cold complete medium for immediate immune cell isolation. The colon was removed, measured, and flushed with ice-cold PBS, with one-third fixed in 4% paraformaldehyde for histological examination, two-thirds snap-frozen for subsequent ELISA, Western Blot, and RNA-sequencing. Bilateral maxillae were dissected free of soft tissue. One side was fixed in 4% paraformaldehyde (4 °C, 48 h) and transferred to EDTA (pH 7.2, 4 °C) for gentle decalcification. Decalcified specimens were processed through graded ethanol, xylene, and paraffin for histological analysis. The contralateral half was immersed in 3% H_2_O_2_ (4 °C, overnight) to remove surface blood, rinsed in 97% alcohol, and stained with 0.1% methylene blue for gross morphometric assessment of alveolar bone loss. All samples were coded to ensure blinding during subsequent analyses. Mice were euthanized by cervical dislocation under deep anesthesia, and death was confirmed by cessation of cardiac and respiratory reflexes.

### Biochemical and immunological analyses

The spleen index (mg/g) was calculated as spleen weight (mg) divided by body weight (g) according to the following formula: spleen index = spleen weight (mg)/body weight (g) to gauge systemic immune status^56^.

Superoxide dismutase (SOD) activity and malondialdehyde (MDA) level were colorimetrically quantified with commercial WST-1 and TBA kits (SOD Detection Kit-WST, Product Number: A001-3; MDA Assay Kit-TBA, Product Number: A003-1-2; Nanjing Jiancheng, China), respectively, following the vendor’s protocols.

Serum and colonic concentrations of IL-1β, IL-6, TNF-α, IL-10, MUC2, and MPO were assessed with enzyme-linked immunosorbent assay (ELISA) kits (Jiangsu Meimian Industrial Co., Ltd.) following the manufacturer’s instructions.

### Disease activity index (DAI)

Body-mass change, fecal consistency, and visible rectal bleeding were monitored daily by two blinded observers^57^. Percentage weight change was computed with respect to Day 0: weight loss (%) = (initial body weight-final body weight)/initial body weight × 100. The DAI was calculated as: DAI = (weight-loss score + stool-consistency score + hematochezia score)/3.

### Histological and histomorphometric analysis

Hematoxylin and eosin (H&E) staining was used to evaluate pathological alterations in both periodontal and colonic tissues.

To quantify alveolar bone resorption, the buccal and palatal surfaces of the second maxillary molar were photographed after 0.1% methylene-blue staining; bone resorption was measured at six standardized points—three each on the buccal and palatal sides of the alveolar crest (mesial, mid, distal)—were measured. Bone resorption was defined as the vertical distance from the alveolar crest to the cementoenamel junction (CEJ), measured perpendicular to the long axis of the tooth. The six measurements were averaged to obtain a single bone loss value per specimen.

Tartrate-resistant acid phosphatase (TRAP) staining (G1492, Solarbio) was employed to identify osteoclasts. The region of interest (ROI) was defined as the periodontal tissue below the furcation and near the root apex of the maxillary second molar, where active bone resorption is most prominent. Multinucleated (≥3 nuclei) TRAP-positive cells were counted per unit area with Image Pro Plus 6.0.

Immunohistochemical detection of tight junction proteins (occludin, dilution 1:200, Proteintech, 27260-1-AP, USA; ZO-1, dilution 1:200, Abcam, ab190085, USA) and the bone remodeling mediator RANKL (dilution 1:200, Proteintech, 23408-1-AP, USA) and OPG (dilution 1:500, Proteintech, AB-3671131, USA) was performed as previously described^56^. Occludin and ZO-1 were analyzed in the distal third of the colon (lower segment), approximately 1 cm proximal to the rectum, whereas RANKL and OPG were analyzed in the alveolar bone adjacent to the root surface of the maxillary second molar.

### Western blotting analysis

Equal amounts of protein, quantified using the BCA protein assay kit, were separated by 12% sodium dodecyl sulfate-polyacrylamide gel electrophoresis (SDS-PAGE) and transferred onto polyvinylidene difluoride (PVDF) membranes. The membranes were blocked with 5% bovine serum albumin (BSA) in Tris-buffered saline for 2 h at room temperature. Following blocking, the blots were incubated overnight at 4 °C with primary antibodies specific to Occludin (1:1000 dilution, Proteintech, 27260-1-AP, USA) and ZO-1 (1:500 dilution, Abcam, ab190085, USA), followed by incubation with appropriate horseradish peroxidase (HRP)-conjugated secondary antibodies. Protein bands were detected using an enhanced chemiluminescence (ECL) detection kit (MilliporeSigma, Burlington, MA, USA), and relative band intensities were quantified using ImageJ software (National Institutes of Health, Bethesda, MD, USA).

### Flow cytometry

The proportions of Th17 and Treg cells were analyzed using flow cytometry. In brief, lymphocytes were isolated from mouse spleens and lymph nodes and washed twice with phosphate-buffered saline (PBS), and subjected to viability staining using the Live/Dead Fixable Near-IR Dead Cell Stain Kit (Thermo Fisher Scientific, L34975, USA). The isolated cells were then transferred to test tubes and incubated with surface antibodies: KO-labeled CD4 (anti-mouse, BD Biosciences, 553046, USA) and EF700-labeled CD25 (anti-mouse, BD Biosciences, 557192, USA). Following another wash with PBS, 1 ml of RPMI 1640 medium (Gibco, USA) containing phorbol 12-myristate 13-acetate (PMA), ionomycin, and monensin (all from MultiSciences, China) was added to each tube. The cells were then incubated at 37 °C for 4 h to stimulate cytokine production. Subsequently, cells were washed, fixed, and permeabilized, followed by intracellular staining with PE-Cy7-labeled IL-17A (anti-mouse, BD Biosciences, 559502, USA), PE-labeled RORγt (anti-mouse, eBioscience, 12-6981-60, USA), and FITC-labeled FOXP3 (anti-mouse, BD Biosciences, 563101, USA) for 60 min at 4 °C. After additional washes, the cells were analyzed using a BD LSR Fortessa flow cytometer (BD Biosciences, USA). Data were processed with FlowJo software (Tree Star Inc., San Carlos, CA).

Gating strategy: The sequential gating hierarchy was performed as follows: (1) lymphocytes were identified based on FSC-A/SSC-A; (2) doublets were excluded using FSC-H vs. FSC-W and SSC-H vs. SSC-W; (3) live cells were gated using viability dye; (4) CD4⁺ T cells were selected from the live cell population; and (5) Th17 and Treg subsets were identified within the CD4⁺ gate (Figure S3). Th17 cells were defined as CD4⁺RORγt⁺IL-17A⁺, and Treg cells were defined as CD4⁺CD25⁺FOXP3⁺.

### RNA isolation and quantitative real-time PCR (qRT-PCR)

Total RNA was extracted from colon tissues with Trizol (Invitrogen, USA) and reverse-transcribed using random primers (Invitrogen, USA). qRT-PCR was subsequently run on a StepOnePlus System (Applied Biosystems, Foster City, USA) with SYBR Green and gene-specific primers (Table S1). The relative expression was calculated by the Ct^2(−ΔΔCt)^ method with GAPDH as endogenous control^58^.

### Saliva and feces 16S rRNA sequencing and bioinformatics analysis

The FastPure Stool DNA Isolation Kit (MJYH, Shanghai, China) was used to extract the total DNA based on the manufacturer’s instructions. The specific primers with barcode were used to amplify the samples’ DNA to enrich the bacterial 16S V3–V4 rRNA regions (V3: 338 F, ACTCCTACGGGAGGCAGCAG-3′ and V4: 806 R, GGACTACHVGGGTWTCTAAT-3′)^59^. The PCR amplification products were then recovered and quantified using Qubit 4.0 fluorometer (Thermo Fisher Scientific, USA) and the PCR Clean-Up Kit (YuHua, Shanghai, China). Subsequently, the purified amplicons were pooled in equimolar amounts and paired-end sequenced on an Illumina Nextseq2000 platform (Illumina, San Diego, USA) according to the standard protocols by Majorbio Bio-Pharm Technology Co., Ltd. (Shanghai, China).

For the statistical analyses of 16S rRNA data, the Pan value was calculated using Usearch software^60^. Alpha-diversity metrics were computed using Mothur, providing insights into the complexity of the microbial communities. PCoA and NMDS were conducted in vegan and mixOmics R packages to visualize the beta-diversity of the samples. To identify bacterial taxa with significant differences in abundance, both phylum and genus levels were examined using Student’s *t*-test, with a significance threshold set at *P* < 0.05. Species clustering was visualized through the R software, facilitating the interpretation of microbial relationships. Additionally, LEfSe was employed to screen for biomarkers across taxonomic ranks from phylum to genus that significantly contributed to group differences. The selection criteria included an LDA score > 4 and *P* < 0.05. Finally, functional prediction analysis of the microbiota was carried out using PICRUSt2 (version 2.2.0), enabling the inference of potential metabolic pathways within the communities. Additionally, Pearson correlation analysis was conducted to evaluate the relationships, whether positive or negative, between differentially abundant genera and physiological indicators. Providing deeper insights into microbial interactions and their possible functional implications.

### LC–MS methods

Metabolomic profiling was carried out on a UHPLC-Q Exactive HF-X system equipped with an ACQUITY HSS T3 column (100 mm × 2.1 mm i.d., 1.8 μm; Waters, USA) at Majorbio Bio-Pharm Technology Co., Ltd. (Shanghai, China). Mobile phase A composed of 0.1% formic acid in water–acetonitrile (2:98, v/v) and phase B of 0.1% formic acid in acetonitrile, delivered at 0.40 mL/min and 40 °C^61^. Raw LC–MS data were processed in Progenesis QI software (v3.0), ānd differential metabolites are identified by variable importance in projection (VIP) > 1.0, *P*-value < 0.05, and fold change (FC) > 1.2. Subsequently, metabolic pathway annotations for these differential metabolites were mapped to KEGG pathways for enrichment analysis.

Furthermore, the top 30 differentially expressed metabolites and genera at the genus level were selected for calculating Pearson correlation coefficients (*R*) and corresponding *P*-values.

### Fecal short-chain fatty acids (SCFAs) detection

SCFAs were quantified by gas chromatography–mass spectrometry (GC–MS) at Shanghai Majorbio Biotechnology Co., Ltd. (Shanghai, China) using an Agilent 8890B-5977B system equipped with an HP-FFAP capillary column (30 m × 0.25 mm × 0.25 μm) and ultrahigh-purity helium (99.999%, 1 mL/min) as carrier gas with a temperature gradient from 80 °C to 120 °C (40 °C/min), then raised to 200 °C at 5 °C/min, hold 3 min at 220 °C^62^. External calibration curves for acetic acid, propanoic acid, isobutyric acid, butanoic acid, isovaleric acid, and valeric acid were constructed for absolute quantification. Spearman correlation analysis was conducted to reveal the correlations between the differential gut microbiota and the three most abundant acids—acetic acid, propanoic acid, and butanoic acid.

### RNA sequencing and data analysis

RNA-seq library construction and sequencing were performed at Shanghai Majorbio Bio-Pharm Technology Co., Ltd. (Shanghai, China) essentially as described by Kong et al.^63^. In brief, total RNA extracted from mouse colon tissue was checked for integrity with an Agilent RNA 6000 Nano Kit on the Agilent Bioanalyzer 2100 system. Double-stranded cDNA synthesized from the total RNA was used for PCR amplification; amplicons were purified with the AMPure XP system and quantified on the Bioanalyzer. Qualified libraries were subsequently paired-end sequenced on the Illumina Novaseq platform.

Paired-end reads were trimmed and filtered using fastp^64^ with default parameters, and subsequently aligned to the reference genome in a strand-specific manner using HISAT2^65^. PCA was applied to visualize sample clustering based on the top 500 most variable genes. Differential expression analysis between groups was performed using DESeq2^66^. Genes exhibiting an adjusted *P*-value < 0.05 and FC ≥ 1.5 were regarded as significant. The clusterProfiler R package was utilized to statistically analyze the enrichment of these differentially expressed genes within KEGG pathways. Finally, to link host transcription to microbial shifts, Spearman correlation was calculated between the differentially abundant intestinal bacterial genera and the selected genes.

### Statistical analysis

Sample size (*n* = 3–8) for each experiment was determined based on preliminary studies and varied across endpoints according to technical feasibility. Post hoc power analysis was performed to verify statistical adequacy by G*Power 3.1.9.7. Effect sizes ranged from Cohen’s *f* = 0.97–5.66 (four-group ANOVA) and Cohen’s *d* = 1.49–8.49 (two-group *t*-test), with power exceeding 80% for almost all outcomes (Tables S2 and S3).

All data were analyzed using SPSS 29.0 software (IBM, USA). Measurement data are presented as mean ± standard deviation (mean ± SD). For data meeting the assumptions of normal distribution and homogeneity of variance, comparisons between two groups were performed using the independent samples *t*-test, while comparisons among multiple groups were conducted using one-way analysis of variance (ANOVA). Statistical significance was set at *P* < 0.05, with *P* < 0.05, *P* < 0.01, and *P* < 0.001 indicating statistically significant differences.

## Supplementary information

Figure S1-S4

Table S1

Table S2

Table S3

Supplementary materials

## Data Availability

The raw sequence data reported in this paper have been deposited in the Genome Sequence Archive (Genomics, Proteomics & Bioinformatics 2025) in the National Genomics Data Center (Nucleic Acids Res 2025), China National Center for Bioinformation / Beijing Institute of Genomics, Chinese Academy of Sciences (GSA: CRA035859, CRA035861, and CRA035787), which are publicly accessible at https://ngdc.cncb.ac.cn/gsa. Additionally, the metabolomics data were submitted to the OMIX, China National Center for Bioinformation / Beijing Institute of Genomics, Chinese Academy of Sciences (https://ngdc.cncb.ac.cn/omix: accession nos. OMIX013857 and OMIX013858).
