# Supplementary material for: Ulcerative colitis-driven gut dysbiosis exacerbates periodontal bone loss through the gut–oral axis /Th17/Treg imbalance
Source: NPJ Biofilms Microbiomes. 2026 May 23;12:154. doi: 10.1038/s41522-026-01015-6 (PMC13473518; doi:10.1038/s41522-026-01015-6)
Supplement: Supplementary file 1 [file 41522_2026_1015_MOESM1_ESM.pdf]

Figure S1

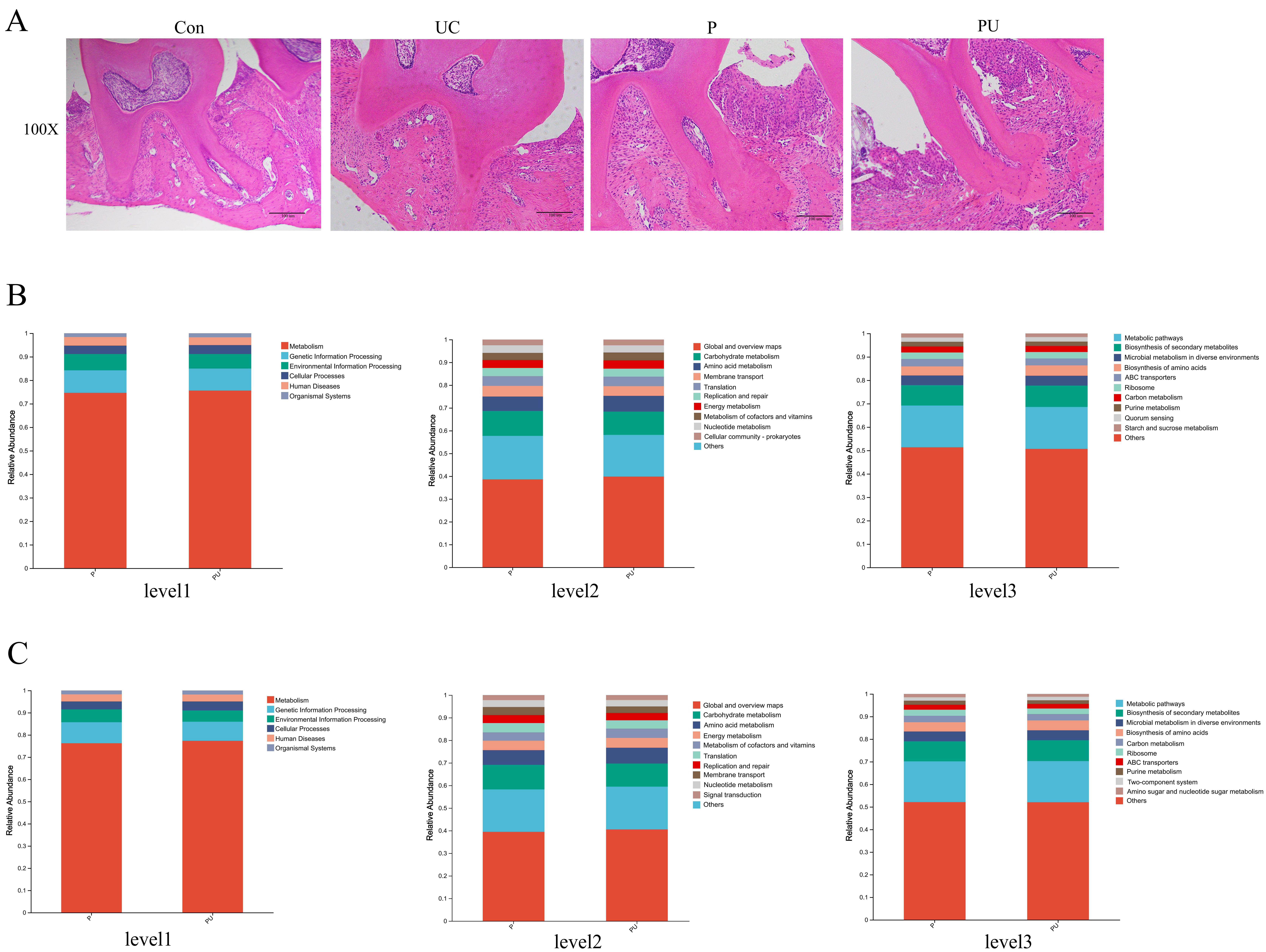

A
Con
UC
P
PU
100X
B
Relative Abundance
Metabolism
Genetic Information Processing
Environmental Information Processing
Cellular Processes
Human Diseases
Organismal Systems
level1
Global and overview maps
Carbohydrate metabolism
Amino acid metabolism
Membrane transport
Translation
Replication and repair
Energy metabolism
Metabolism of cofactors and vitamins
Nucleotide metabolism
Cellular community - prokaryotes
Others
level2
Metabolic pathways
Biosynthesis of secondary metabolites
Microbial metabolism in diverse environments
Biosynthesis of amino acids
ABC transporters
Ribosome
Carbon metabolism
Purine metabolism
Quorum sensing
Starch and sucrose metabolism
Others
level3
C
Metabolism
Genetic Information Processing
Environmental Information Processing
Cellular Processes
Human Diseases
Organismal Systems
level1
Global and overview maps
Carbohydrate metabolism
Amino acid metabolism
Energy metabolism
Metabolism of cofactors and vitamins
Translation
Replication and repair
Membrane transport
Nucleotide metabolism
Signal transduction
Others
level2
Metabolic pathways
Biosynthesis of secondary metabolites
Microbial metabolism in diverse environments
Biosynthesis of amino acids
Carbon metabolism
Ribosome
ABC transporters
Purine metabolism
Two-component system
Amino sugar and nucleotide sugar metabolism
Others
level3

Figure S2

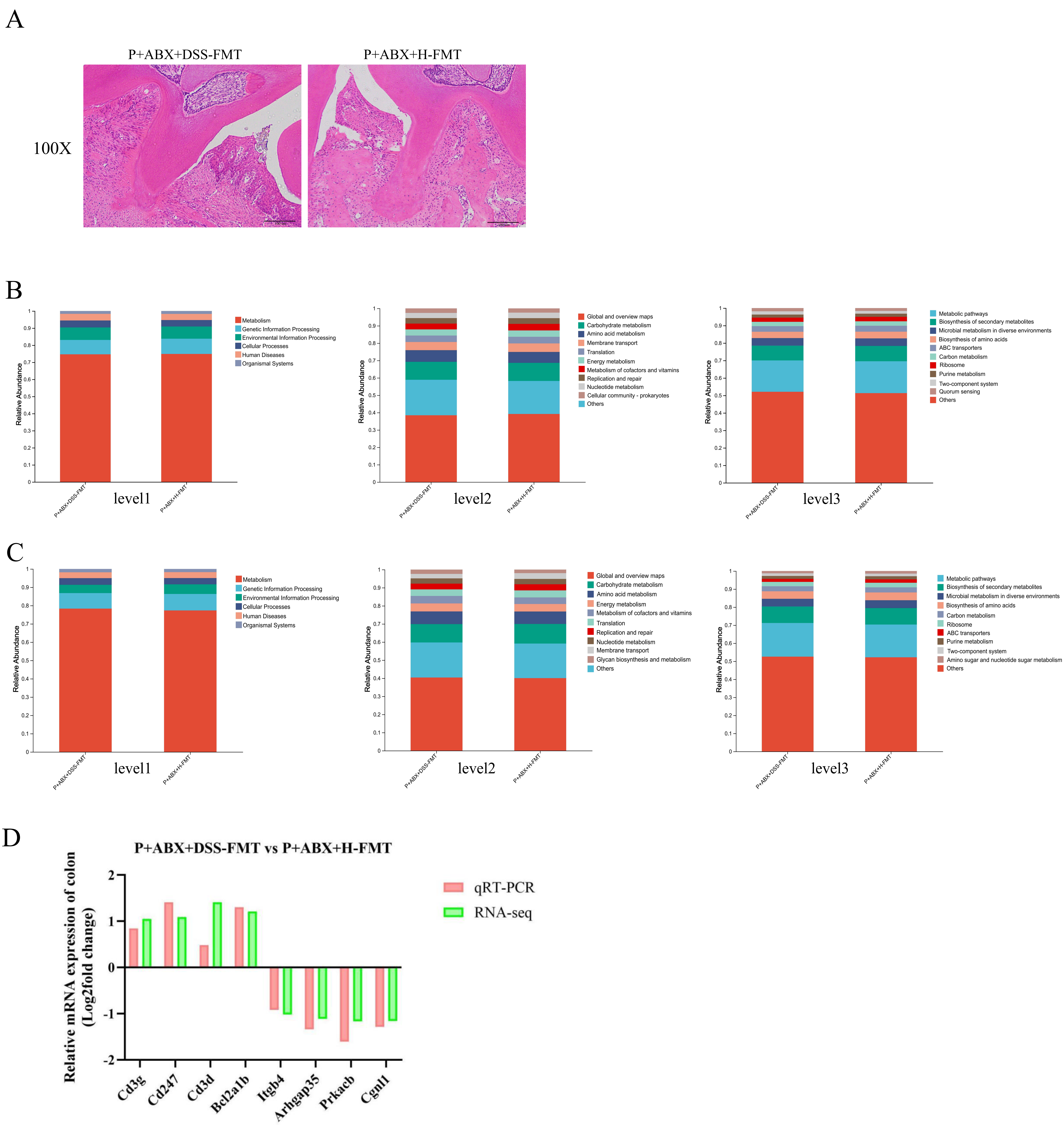

A
P+ABX+DSS-FMT
P+ABX+H-FMT
100X
B
Relative Abundance
level1
level2
level3
P+ABX+DSS-FMT
P+ABX+H-FMT
Metabolism
Genetic Information Processing
Environmental Information Processing
Cellular Processes
Human Diseases
Organismal Systems
Global and overview maps
Carbohydrate metabolism
Amino acid metabolism
Membrane transport
Translation
Energy metabolism
Metabolism of cofactors and vitamins
Replication and repair
Nucleotide metabolism
Cellular community - prokaryotes
Others
Metabolic pathways
Biosynthesis of secondary metabolites
Microbial metabolism in diverse environments
Biosynthesis of amino acids
ABC transporters
Carbon metabolism
Ribosome
Purine metabolism
Two-component system
Quorum sensing
Others
C
Relative Abundance
level1
level2
level3
P+ABX+DSS-FMT
P+ABX+H-FMT
Metabolism
Genetic Information Processing
Environmental Information Processing
Cellular Processes
Human Diseases
Organismal Systems
Global and overview maps
Carbohydrate metabolism
Amino acid metabolism
Energy metabolism
Metabolism of cofactors and vitamins
Translation
Replication and repair
Nucleotide metabolism
Membrane transport
Glycan biosynthesis and metabolism
Others
Metabolic pathways
Biosynthesis of secondary metabolites
Microbial metabolism in diverse environments
Biosynthesis of amino acids
Carbon metabolism
Ribosome
ABC transporters
Purine metabolism
Two-component system
Amino sugar and nucleotide sugar metabolism
Others
D
P+ABX+DSS-FMT vs P+ABX+H-FMT
Relative mRNA expression of colon
(Log2fold change)
qRT-PCR
RNA-seq
Cd3g
Cd247
Cd3d
Bcl2a1b
Itgb4
Arhgap35
Prkacb
Cgnl1

Figure S3

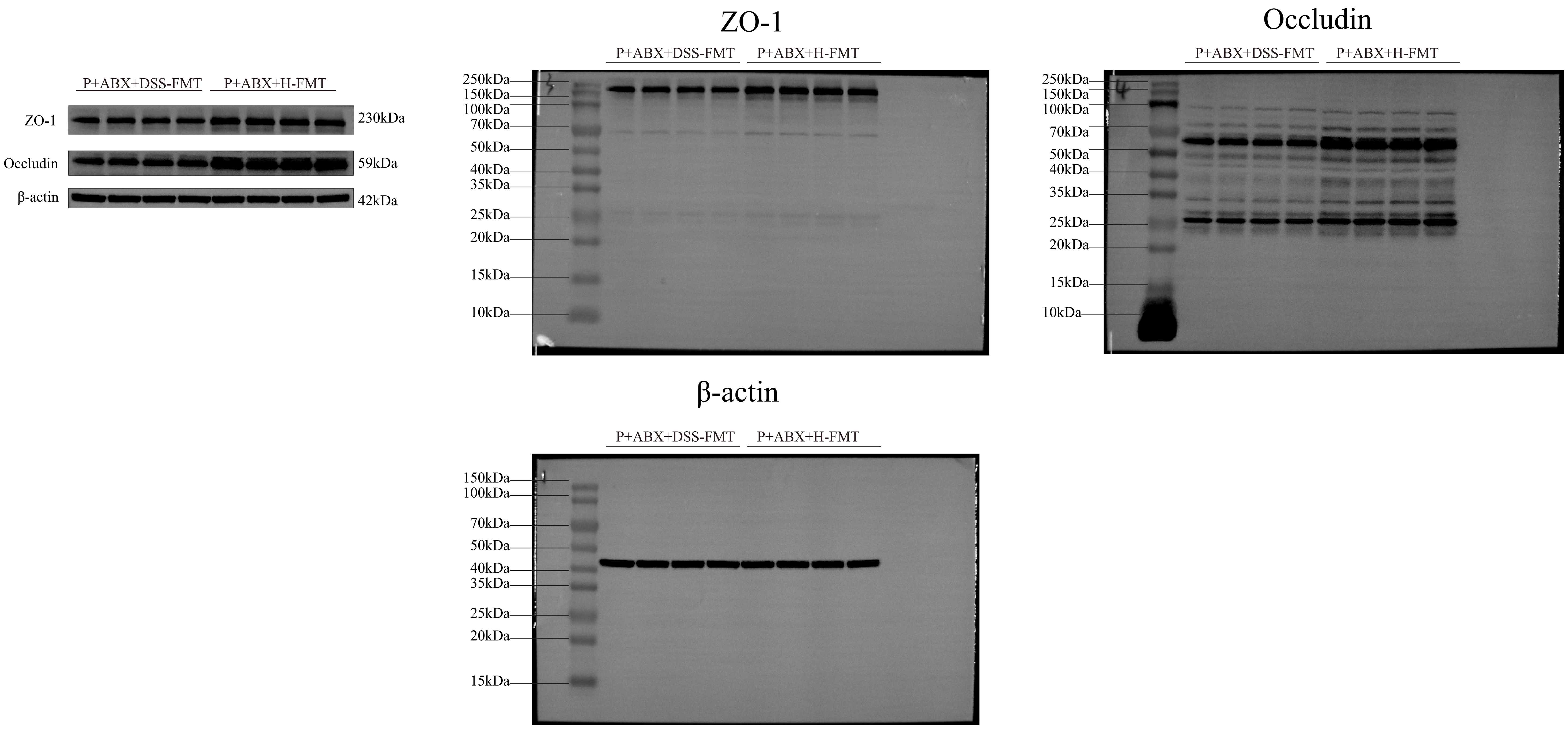

P+ABX+DSS-FMT
P+ABX+H-FMT
ZO-1
230kDa
Occludin
59kDa
β-actin
42kDa
ZO-1
P+ABX+DSS-FMT
P+ABX+H-FMT
250kDa
150kDa
100kDa
70kDa
50kDa
40kDa
35kDa
25kDa
20kDa
15kDa
10kDa
Occludin
P+ABX+DSS-FMT
P+ABX+H-FMT
250kDa
150kDa
100kDa
70kDa
50kDa
40kDa
35kDa
25kDa
20kDa
15kDa
10kDa
β-actin
P+ABX+DSS-FMT
P+ABX+H-FMT
150kDa
100kDa
70kDa
50kDa
40kDa
35kDa
25kDa
20kDa
15kDa

Figure S4

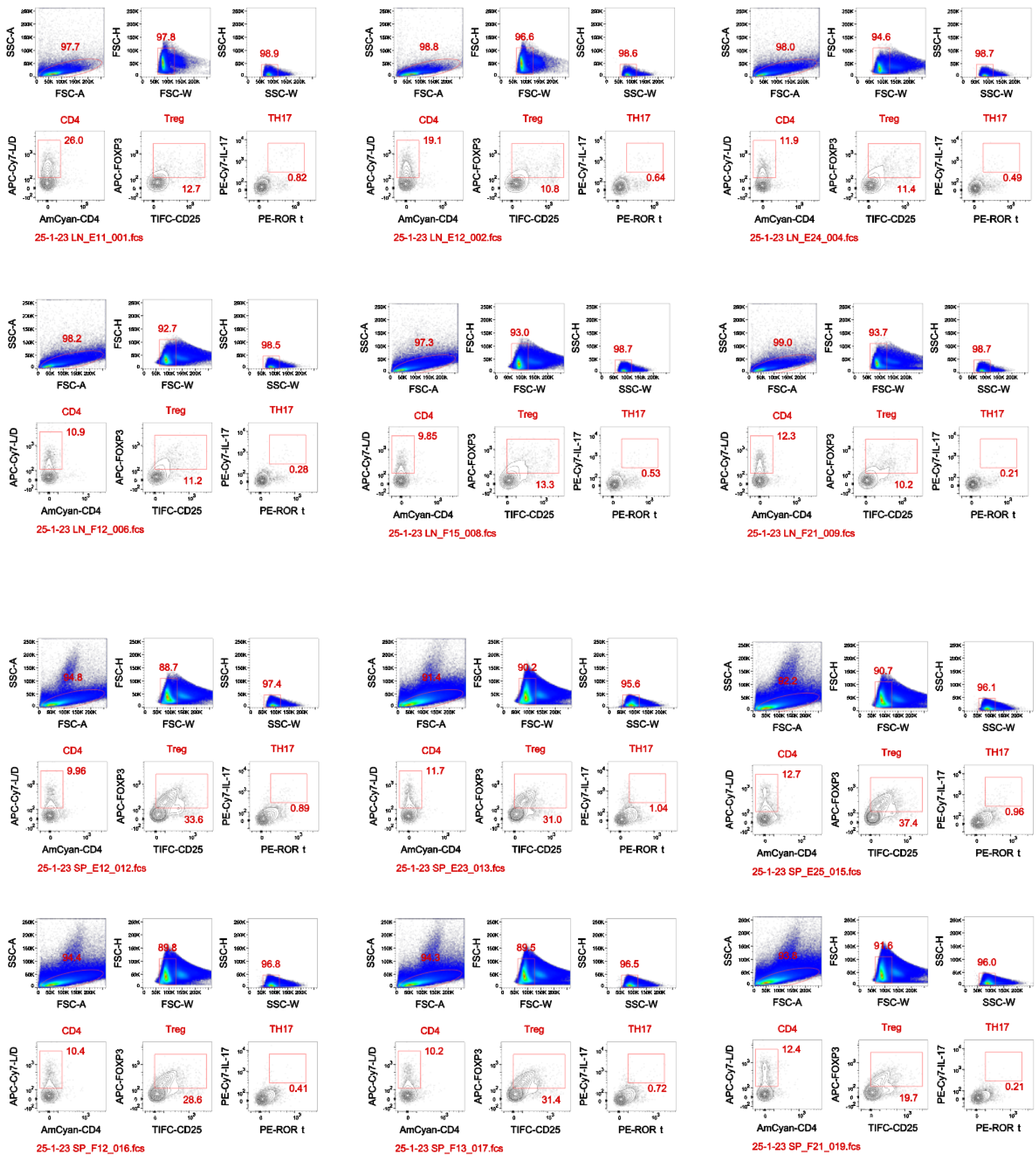
SSC-A
FSC-A
FSC-H
FSC-W
SSC-H
SSC-W
CD4
Treg
TH17
APC-Cy7-L/D
AmCyan-CD4
APC-FOXP3
TIFC-CD25
PE-Cy7-IL-17
PE-ROR t
97.7
97.8
98.9
26.0
12.7
0.82
25-1-23 LN_E11_001.fcs
98.8
96.6
98.6
19.1
10.8
0.64
25-1-23 LN_E12_002.fcs
98.0
94.6
98.7
11.9
11.4
0.49
25-1-23 LN_E24_004.fcs
98.2
92.7
98.5
10.9
11.2
0.28
25-1-23 LN_F12_006.fcs
97.3
93.0
98.7
9.85
13.3
0.53
25-1-23 LN_F15_008.fcs
99.0
93.7
98.7
12.3
10.2
0.21
25-1-23 LN_F21_009.fcs
94.8
88.7
97.4
9.96
33.6
0.89
25-1-23 SP_E12_012.fcs
91.4
90.2
95.6
11.7
31.0
1.04
25-1-23 SP_E23_013.fcs
92.2
90.7
96.1
12.7
37.4
0.96
25-1-23 SP_E25_015.fcs
94.4
89.8
96.8
10.4
28.6
0.41
25-1-23 SP_F12_016.fcs
94.3
89.5
96.5
10.2
31.4
0.72
25-1-23 SP_F13_017.fcs
93.8
91.6
96.0
12.4
19.7
0.21
25-1-23 SP_F21_019.fcs
