# Supplementary material for: Ulcerative colitis-driven gut dysbiosis exacerbates periodontal bone loss through the gut–oral axis /Th17/Treg imbalance
Source: NPJ Biofilms Microbiomes. 2026 May 23;12:154. doi: 10.1038/s41522-026-01015-6 (PMC13473518; doi:10.1038/s41522-026-01015-6)
Supplement: Supplementary file 2 [file 41522_2026_1015_MOESM5_ESM.docx]

**Supplementary materials**：

**Table S1**. Sequences for primers used in qRT-PCR.

**Table S2**. Post Hoc Power analysis for One-Way ANOVA comparing Con, UC, P, and PU groups.

**Table S3**. Post Hoc Power analysis for independent-samples *t*-Test comparing FMT recipient groups (P+ABX+DSS-FMT vs. P+ABX+H-FMT).

**Figure S1**. (A) Representative H&E-stained periodontal tissues images surrounding the maxillary second molar in the Con, UC, P, and PU groups (100× magnification, N=3/group, Scale bar: 100 μm). (B, C) PICRUSt2-based functional profiling of oral (B) and gut (C) microbiota in the P and PU groups across KEGG levels1, 2, and 3.

**Figure S2**. (A) Representative H&E stained periodontal tissues images surrounding the maxillary second molar in the P+ABX+DSS-FMT and P+ABX+H-FMT groups (100× magnification, N=3/group, Scale bar:100 μm). (B, C) PICRUSt2-based functional profiling of oral (B) and gut (C) microbiota in the P+ABX+DSS-FMT and P+ABX+H-FMT groups across KEGG levels1, 2, and 3 (D) RNA Isolation and Quantitative Real-Time PCR (qRT-PCR) validation of differentially expressed genes in colonic tissues (N=3/group).

**Figure S3**. Original Western blot images of occludin and ZO-1 in colonic tissues in FMT recipients.

**Figure S4**. Gating strategy for flow cytometry analysis of Th17 and Treg cells. Lymphocytes were first gated based on FSC-A and SSC-A, followed by doublet exclusion (FSC-H vs FSC-W) and live/dead discrimination. CD4⁺ T cells were then identified, and Th17 (CD4⁺IL-17A⁺RORγt^+^) and Treg (CD4⁺CD25⁺Foxp3⁺) cells were defined within the CD4⁺ population. Numbers indicate the percentage of cells within the parent gate.
